# Synthetically accessible de novo design using reaction vectors: Application to PARP1 inhibitors**

**DOI:** 10.1002/minf.202300183

**Published:** 2024-02-06

**Authors:** Gian Marco Ghiandoni, Stuart R. Flanagan, Michael J. Bodkin, Maria Giulia Nizi, Albert Galera‐Prat, Annalaura Brai, Beining Chen, James E. A. Wallace, Dimitar Hristozov, James Webster, Giuseppe Manfroni, Lari Lehtiö, Oriana Tabarrini, Valerie J. Gillet

**Affiliations:** ^1^ Information School University of Sheffield Regent Court, 211 Portobello Sheffield S1 4DP UK; ^2^ Evotec (U.K.) Ltd 114 Innovation Drive, Milton Park Abingdon OX14 4RZ UK; ^3^ Department of Chemistry University of Sheffield Dainton Building, Brook Hill Sheffield S3 7HF UK; ^4^ Department of Pharmaceutical Sciences University of Perugia 06123 Perugia Italy; ^5^ Faculty of Biochemistry and Molecular Medicine & Biocenter Oulu University of Oulu Oulu FI-90014 Finland; ^6^ Department of Biotechnology, Chemistry and Pharmacy University of Siena I-53100 Siena Italy

**Keywords:** de novo drug design, PARP1 inhibitors, pharmaceuticals, reaction informatics, synthetic accessibility

## Abstract

*De novo* design has been a hotly pursued topic for many years. Most recent developments have involved the use of deep learning methods for generative molecular design. Despite increasing levels of algorithmic sophistication, the design of molecules that are synthetically accessible remains a major challenge. Reaction‐based *de novo* design takes a conceptually simpler approach and aims to address synthesisability directly by mimicking synthetic chemistry and driving structural transformations by known reactions that are applied in a stepwise manner. However, the use of a small number of hand‐coded transformations restricts the chemical space that can be accessed and there are few examples in the literature where molecules and their synthetic routes have been designed and executed successfully. Here we describe the application of reaction‐based *de novo* design to the design of synthetically accessible and biologically active compounds as proof‐of‐concept of our reaction vector‐based software. Reaction vectors are derived automatically from known reactions and allow access to a wide region of synthetically accessible chemical space. The design was aimed at producing molecules that are active against PARP1 and which have improved brain penetration properties compared to existing PARP1 inhibitors. We synthesised a selection of the designed molecules according to the provided synthetic routes and tested them experimentally. The results demonstrate that reaction vectors can be applied to the design of novel molecules of biological relevance that are also synthetically accessible.

## Graphical abstract

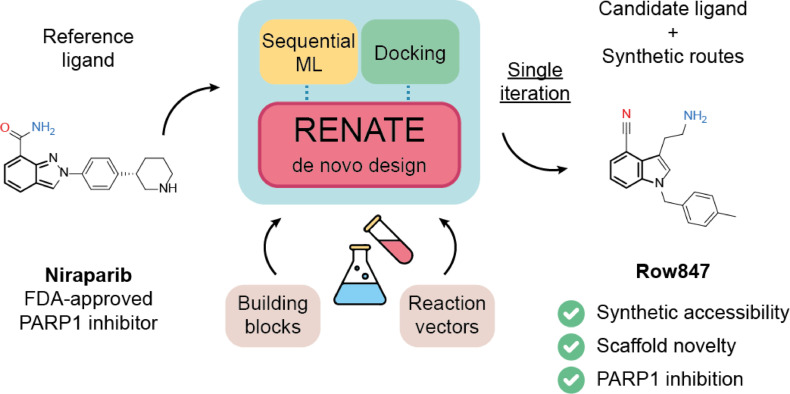

## INTRODUCTION

*De novo* design techniques were first proposed around 30 years ago as a way of accelerating the drug discovery process with many different approaches developed over time. Key issues in *de novo* design are exploring the enormous search space of drug‐like chemical entities effectively while ensuring that the designed compounds are biologically relevant and synthetically accessible [1]. Early approaches were agnostic of synthesis and as a consequence their application was limited [2, 3, 4]. They were later replaced by rule‐based approaches whereby modifications made to starting structures were based on a small number of hand‐coded transformation rules [5, 6]. While these approaches lead to compounds that are more likely to be synthesisable, the use of pre‐defined rules limits the extent of the chemical space that can be explored. Recently, a number of deep generative methods have been developed for *de novo design*, and while these provide data‐driven approaches to promote the search for novel compounds, they typically do not account for synthesis explicitly [5, 6, 7, 8, 9, 10, 11, 12, 13, 14, 15, 16]. A more sophisticated approach was also described recently in which a generative deep learning model was coupled with a rule‐based filter that was used to select compounds compatible with an automated synthesis platform. This method was successful in linking *de novo* design and compound synthesis into an automated workflow; however, it was limited to just 17 synthetic rules and to one‐step syntheses both of which limit the ability of the method to explore diverse areas of chemical space [17].

We have developed a data‐driven approach to the design of novel, synthetically accessible molecules which we refer to as reaction vector‐based *de novo* design [18, 19]. Reaction vectors are derived automatically from databases of reactions so that the available transformations are not limited to a predefined set of rules but are driven by a user‐defined database of reactions. The core of our approach to *de novo* design is a structure generation module, which takes reaction vectors and a database of reagents and applies these to input molecules to generate novel products for which synthetic routes can be provided based on literature precedents. The structure generation module can support different *de novo* design strategies such as “iterative forward synthesis” which starts with a key fragment to which different fragments are added in each iteration. However, this strategy rapidly leads to a combinatorial explosion of possible molecules. We have recently described the integration of the structure generator into an “inside‐out” approach to *de novo* design in a tool called RENATE (REtrosynthetic desigN using reAcTion vEctors) [20]. The starting point is one or more known compounds of interest each of which is fragmented using retrosynthetic rules. A search is then made for similar reagents for each of the resulting fragments and these are combined *in silico* by the structure generator using reaction vectors and external reagents. The approach is similar in concept to Flux and COLIBREE but with a number of important differences [21, 22]. The most significant difference is that the forward construction is driven by known reactions and available reagents so that synthetic routes are provided for the novel compounds. RENATE was previously validated on retrospective design by showing that it could reproduce known drugs and propose meaningful synthetic pathways for them [20]. Here we demonstrate the prospective application of RENATE to the *de novo* design, synthesis and experimental validation of molecules that meet multiple objectives. The study is focused on ADP‐ribosyltransferase PARP1 [23], a nuclear enzyme that has critical involvement in DNA the repair of single‐strand breaks, as proof‐of‐concept. PARP1 is primarily identified as a target in oncology yet recent studies have also suggested it as a potential target against ageing and neurodegenerative diseases such as Alzheimer's disease and Parkinson's disease [24, 25, 26]. However, the brain availability of PARP1 inhibitors is often limited by their low lipophilicity which reduces their ability to cross the blood‐brain barrier and by their affinity for P‐glycoprotein (Pgp) or breast cancer resistance protein (BCRP), which are efflux transporters expressed at the apical membrane of the blood‐brain barrier (BBB).

We used a set of known PARP1 inhibitors as reference ligands for the design. These were fragmented and new molecules were generated *in silico* using reaction vectors derived from the US Pharmaceutical Patents Database and reagents from Enamine [27]. The building blocks/reagents were selected using pharmacophore fingerprint similarity to the fragments from the known inhibitors, and the top‐scoring molecules were selected at each step using a series of machine learning models designed to predict: PARP1 binding; low substrate affinity for Pgp and BCRP, respectively; and good BBB penetration. Following an assessment of the output molecules for the presence of reactive and undesirable groups, the top scoring products were docked against PARP1 and a subset was synthesised based on the synthetic routes proposed by RENATE. Following the synthesis, the compounds were tested against PARP1 in an enzymatic assay. The apparent BBB permeability of two selected compounds was finally measured in a Parallel Artificial Membrane Permeability Assay (PAMPA).

## PARP1 AS A TARGET

**Figure 1:**
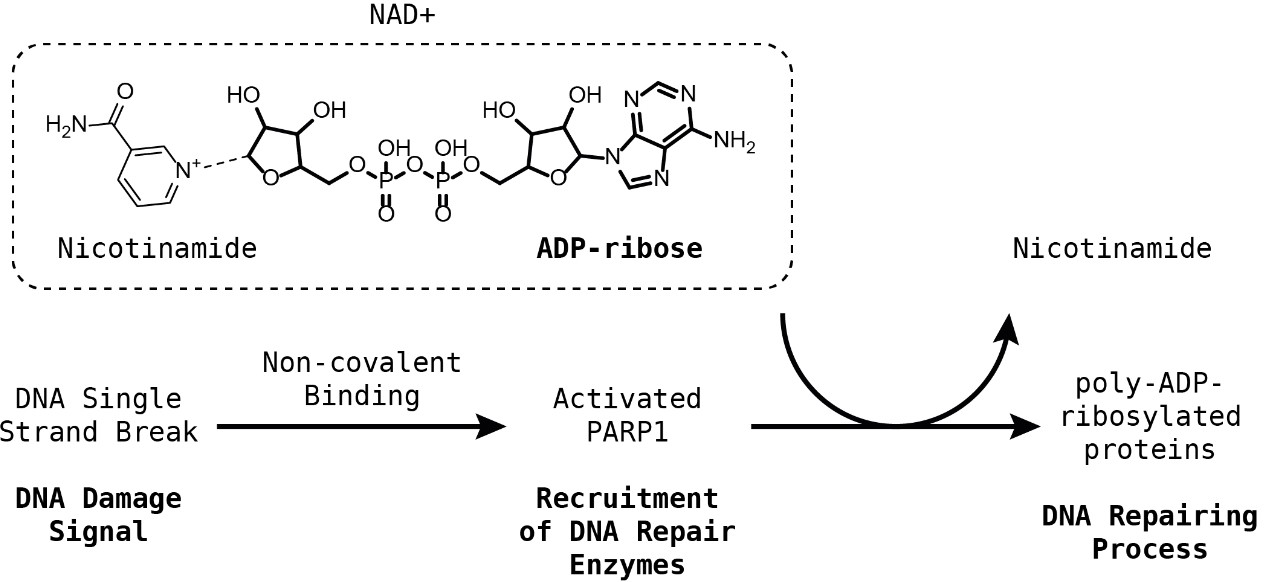
DNA repairing mechanism mediated by PARP1: a DNA single‐strand break activates PARP1 via non‐covalent recognition. The activated PARP1 recruits a series of enzymes responsible for DNA repair via the covalent addition of poly‐ADP‐ribose. The recruited enzymes repair the damaged DNA strand.

PARP1 binds to damaged DNA and promotes the recruitment of repair enzymes through the generation of poly‐ADP‐ribose, which PARP1 attaches to itself and other proteins. PARP1 modifies proteins via the covalent addition of ADP‐ribose and elongates it sequentially to become poly‐ADP‐ribose using NAD^+^ as the donor of ADP‐ribose as shown in Figure 1 [28, 29]. PARP1 plays fundamental roles in other biological processes such as cell proliferation, differentiation, and apoptosis [30]. Upregulation of PARP1 is observed in cancers with BRCA gene defects and has been shown to enhance the resistance of cancers to DNA‐damaging therapies, hence making its inhibition an attractive field of study in pharmaceutical research [31]. The upregulation of PARP1 also leads to a drastic reduction of NAD^+^ levels, affecting ATP production and cell functions, which can lead to the development of other conditions such as diabetes, neurodegenerative diseases, and viral infections [32, 33]. Most PARP1 small molecule inhibitors are designed to act as NAD^+^ mimetics to produce interactions analogous to those between the nicotinamide and the enzyme in order to block the substrate binding site of PARP1.

More than 50 years of effort in medicinal chemistry research have led to the FDA approval of four PARP1 inhibitors, namely, Olaparib (2014), Rucaparib (2016), Niraparib (2017), and Talazoparib (2018). These drugs have currently been approved as chemotherapeutics, in particular against BRCA‐mutated ovarian, fallopian tube, primary peritoneal, pancreatic, prostate and breast cancers [34]. Other PARP1 inhibitors such as Veliparib, Fluzoparib, and Pamiparib are in clinical development [35, 36, 37]. In addition, there are more than 60 studies registered on https://clinicaltrials.gov/ where PARP1 inhibitors are involved [38].

Beyond the role of PARP1 in oncology, emerging research has indicated its potential as a therapeutic target against Alzheimer's disease and Parkinson's disease, which have been shown to upregulate PARP1 and lead to neuroinflammation, mitochondrial dysfunction, and autophagy dysregulation [24, 25]. Hence, the inhibition of PARP1 has also been suggested to maintain the function of the brain and contribute to life span extension [26]. However, the effectiveness of the currently approved PARP1 inhibitors has been shown to be significantly reduced by their poor brain permeability, which makes them unsuitable as neurotherapeutics [39, 40, 41, 42]. Therefore, the development of PARP1 inhibitors with suitable potency and PK profile including high brain permeability and low affinity towards efflux transporters is strongly desired to extend the application of this class of inhibitors against brain cancers and neurodiseases [35, 36, 37, 43, 44].

## RESULTS AND DISCUSSION

### PARP1 structural and ligand data

**Figure 2:**
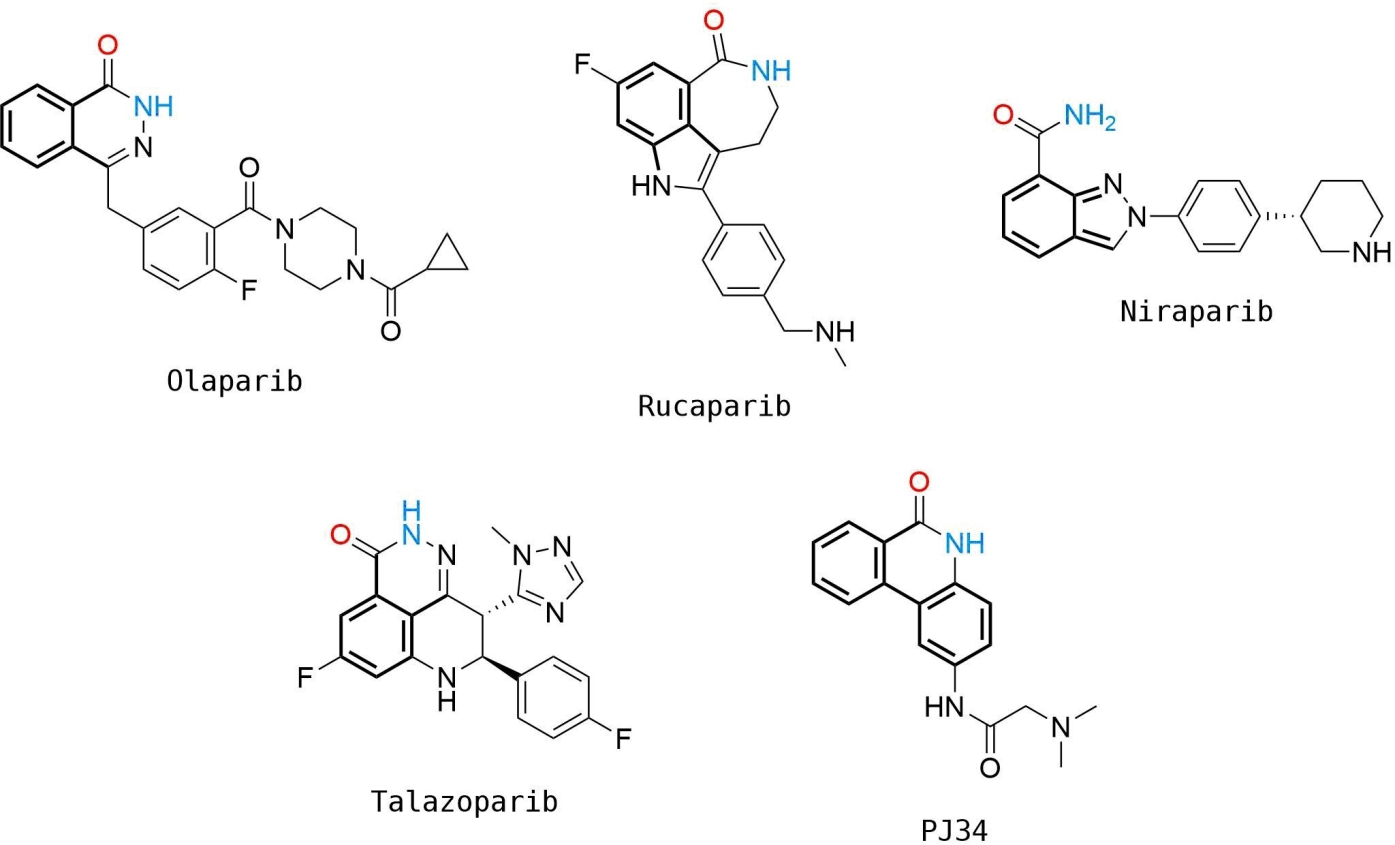
The structures of PARP1 inhibitors from the selected crystal complex structures. These compounds show similar interactions within the receptor and the atoms involved in hydrogen bonding as donors and acceptors are coloured in blue and red, respectively, with substructures involved with π–π stacking interactions shown in bold.

**Figure 3:**
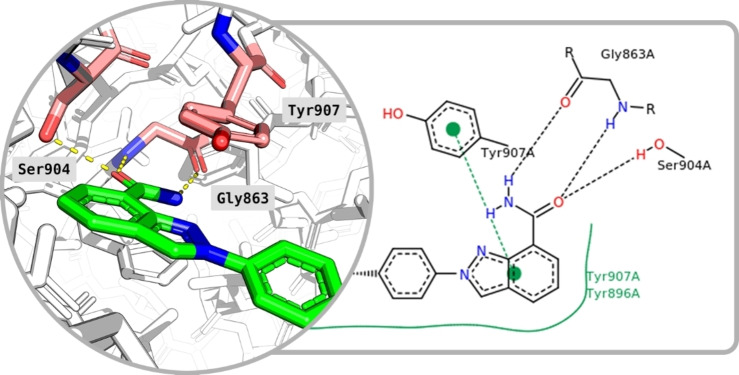
PARP1 3D (left) and 2D (right) key residue interactions with Niraparib. Yellow and black dashed lines indicate hydrogen bonds in 3D and 2D representations, respectively. Green solid lines show hydrophobic interactions and green dashed lines show π–π stacking interactions in the 2D diagram. The 2D diagram was obtained from the PDB.

*Homo sapiens* PARP1 crystal structures in complex with the FDA‐approved drugs, Niraparib (PDB ID: 4R6E–2.2 Å), Talazoparib (PDB ID: 4UND–2.2 Å), Olaparib (PDB ID: 5DS3–2.6 Å), Rucaparib (PDB ID: 4RV6–3.19 Å), and a second‐generation inhibitor, PJ34 (PDB ID: 4UXB–3.22 Å) were retrieved from the Protein Data Bank (PDB) (Figure 2). A detailed representation of Niraparib and its interactions with the enzyme are described in Figure 3.

**Figure 4:**
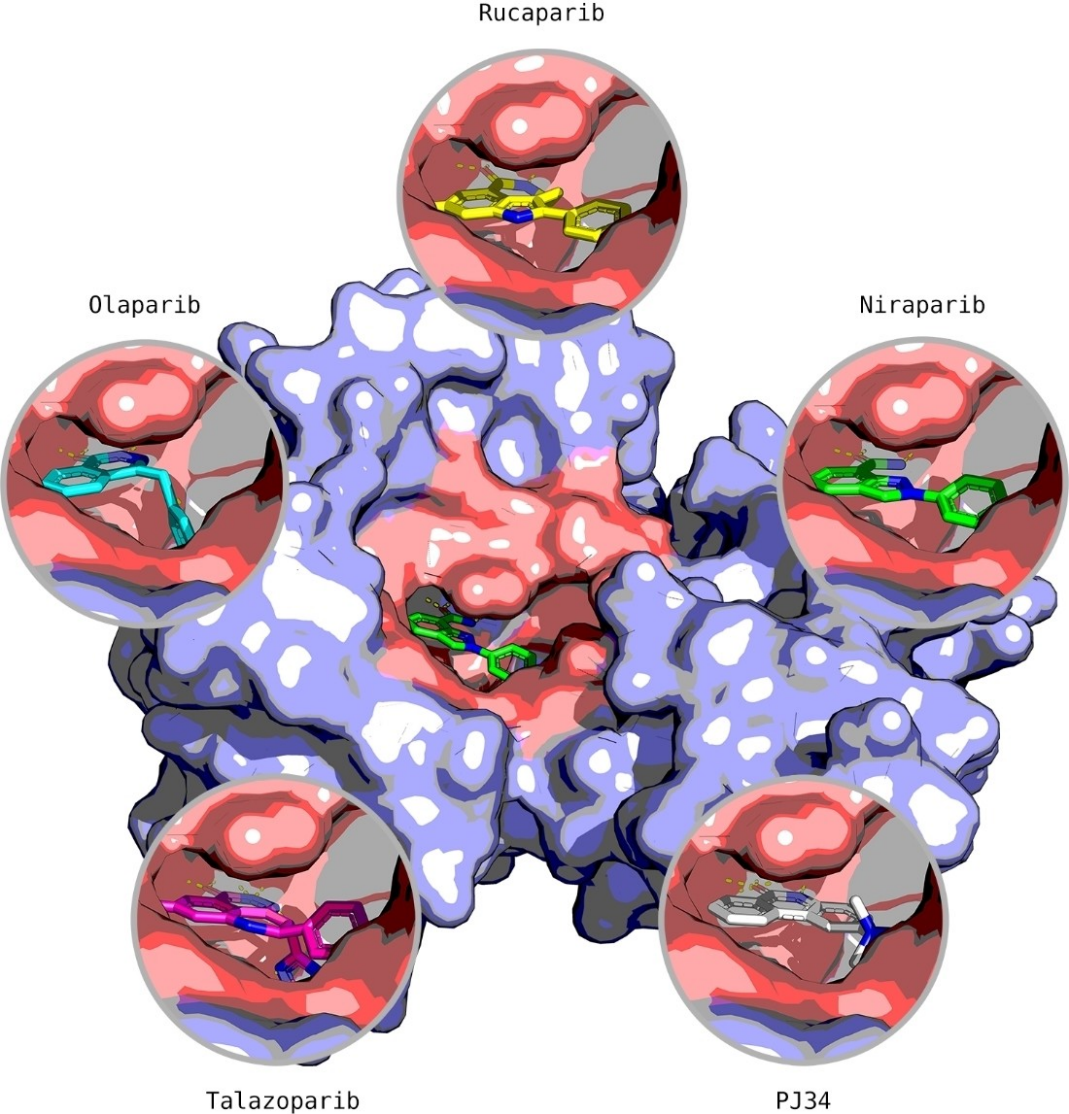
The selected compounds interacting within the binding pocket (pink) of the PARP1 catalytic domain (light purple). The compounds show similar orientation and binding in the pocket.

The selected crystal structures show the three interacting residues that are conserved across the ligands: Gly863 and Ser904, which are responsible for the formation of three hydrogen bonds while Tyr907 produces π–π stacking interactions with the electron‐dense areas of the inhibitors. These residues are also responsible for the interaction with the natural substrate NAD^+^ [45, 46, 47, 48]. The superimposition of all five protein structures produced an excellent structural alignment of the residues in the catalytic domain, with an average root‐mean‐square deviation (RMSD) of 0.543 Å, hence suggesting the suitability of the crystals for cross‐docking. A rendering of the selected compounds aligned within the PARP1 catalytic domain is reported in Figure 4, which shows that the ligands assume similar binding inside the receptor.

### De novo design and compound selection

The five PARP1 inhibitors were processed to yield their key fragments, which in turn were used to search for new building blocks which were combined by RENATE to design new products. The software was configured using a data set of commercial reagents and a reaction vector database, which are described in the Experimental Section. The parameters used in RENATE and the results from the fragmentation are reported in the Supporting Information (Sections S3 and S4). The design was driven by scoring the generated compounds on their predicted PARP1 pIC_50_, Pgp and BCRP substrate inactivity, and BBB+ character. The number of compounds generated from each experiment and their enumerated stereoisomers are reported in Table 1. Note that RENATE produces flat structures, and these must be stereo enumerated to be docked correctly.

**Table 1:** The number of flat and enumerated compounds retained from each design experiment. Note that the Rucaparib and Niraparib experiments were run using two different configurations to produce more candidates. The parameters used in each experiment are reported in the Supporting Information.

Compound	Experiment #	Flat structures	Enumerated structures
Olaparib	1	1,000	1,354
Rucaparib	1	678	769
Rucaparib	2	477	559
Niraparib	1	1,000	1,174
Niraparib	2	1,000	1,064
Talazoparib	1	990	1,339
PJ34	1	1,000	1,694

The fact that Rucaparib and Talazoparib did not reach the maximum number of final products allowed by the design parameters can be explained by the complexity of their chemical motifs which have fewer chances to match the reaction centres in the reaction vector database. These results are in agreement with the principles of reaction vector‐based design: the structural characteristics of the starting materials and reagents affect the number of applicable reaction vectors, which in turn affects the number of products [19].

The final products were then docked against PARP1 with ten poses generated per compound. The docked compounds were inspected manually to identify promising candidates. This was done by sorting compounds on their scores and prioritising the selection of candidates based on the quality of their interactions with the key residues and the number of consistent poses showing valid interactions. Specifically, compounds were selected that showed consistent poses that were qualitatively similar to those of their reference ligands, and which exhibited valid hydrogen‐bond interactions with Gly863 and Ser904 (i. e., a bond distance between 2.5 and 3.0 Å and angle recognised by GOLD as compatible with that of the hydrogen bond). These manual selection criteria were defined due to the difficulty of algorithmically discriminating candidates from other top‐scoring compounds, which, although they generally presented physically meaningful poses and interactions within the binding site, did not possess characteristics similar to their reference ligands. A total number of 20 compounds was selected for synthesis. They are reported in the Supporting Information (Section S5) with their predicted activities, pose consistencies, and average and standard deviations of the binding scores across poses. The selected compounds were predicted to have micromolar pIC_50_, classified as non‐binders of Pgp and BCRP, and to have BBB+ character. In addition, they were all considered to be novel compounds due to their absence from two known suppliers (https://emolecules.com and https://molport.com) and due to no data being available on them in the PubChem and ChEMBL databases (October 2022).

**Figure 5:**
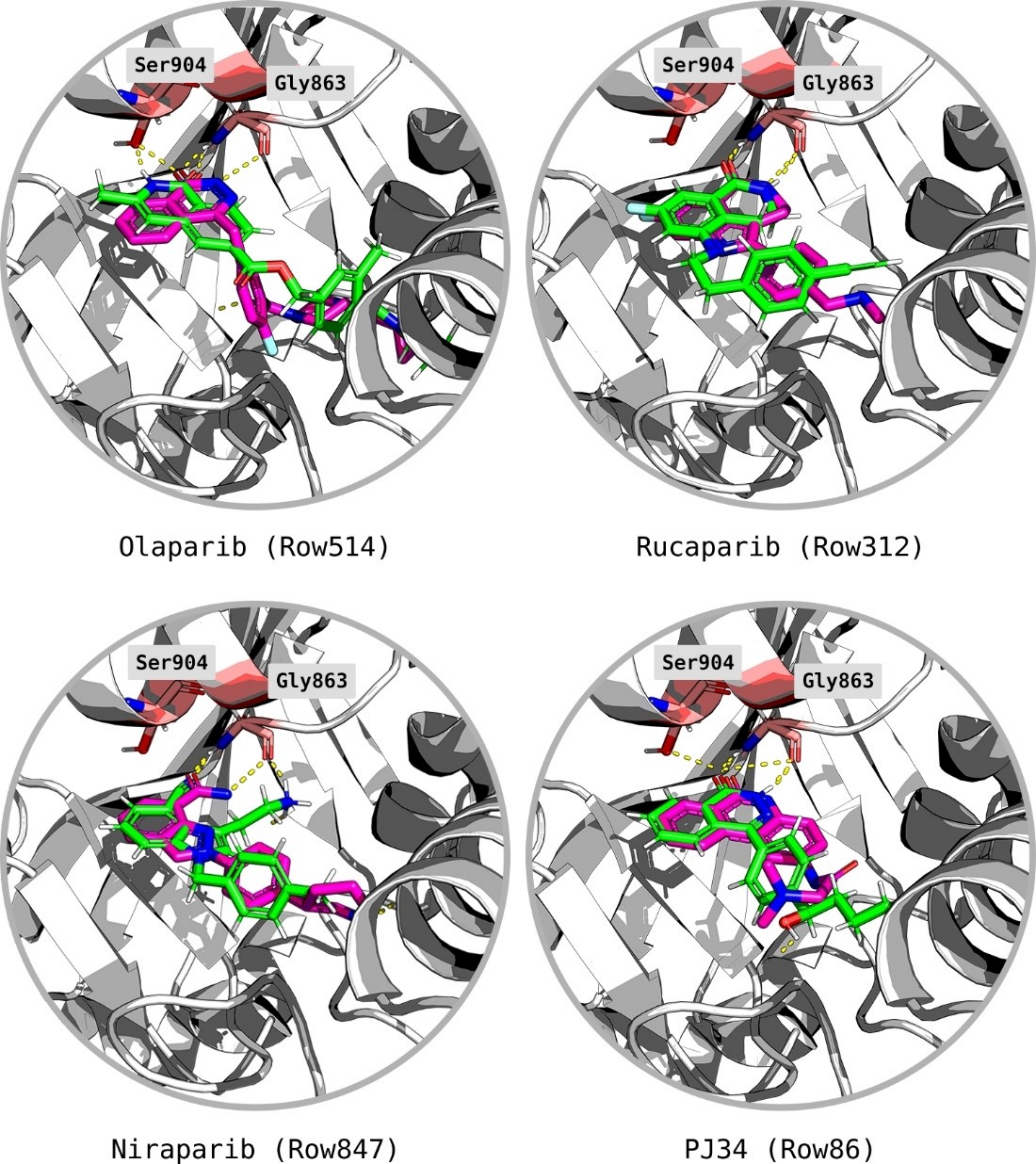
Overlap between candidates (e. g., Row514) (green) and reference ligands (e. g., Olaparib) (purple). The residue Tyr907 is hidden to ease the view of the poses. Hydrogen‐bond interactions between the protein and ligands are displayed in yellow. In particular, the interactions of the ligands with the key residues Ser904 and Gly863 are displayed.

Figure 5 describes some examples of candidates and reference ligands in the binding pocket, which, despite their low 2D pairwise similarity, show good overlap and similar interactions with the key residues. In particular, Olaparib and Row514 (similarity 0.27 using Morgan 1024‐bit fingerprints (Radius 2) and the Tanimoto metric) both have a bicyclic scaffold, but they differ significantly in terms of functionalities and connections. Niraparib and Row847 (similarity 0.22) have different functionalization around the central core but assume similar orientations. Rucaparib and Row312 (similarity 0.40) are also diverse since Rucaparib has a three‐ring motif. A similar outcome is shown for PJ34 and Row86 (similarity 0.24) since PJ34 also has three fused rings, whereas Row86 was designed with a two‐ring scaffold connected to an additional aromatic ring, which was found later in the patent literature [49]. These results show that, in both the Rucaparib and PJ34 cases, RENATE performed scaffold hopping leading to the design of compounds with potential affinity towards PARP1 (i. e., by incorporating motifs present in annotated compounds into newly designed structures). Note that the data used to train the PARP1 activity model did not contain any compounds with the motifs proposed for Row847. This result suggests that RENATE can be used to propose novel scaffolds.

**Figure 6:**
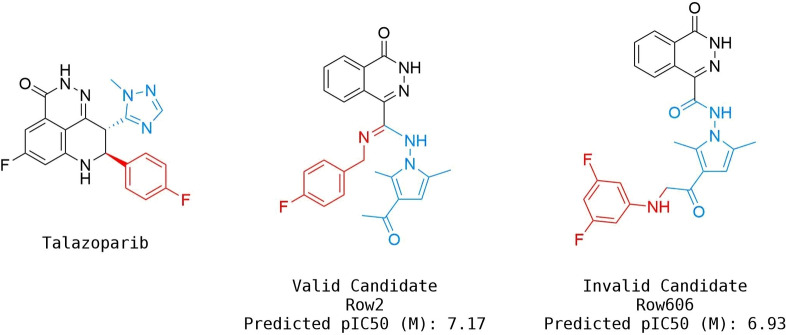
Examples of valid and invalid candidates designed by RENATE using Talazoparib as a reference (A, B, and C fragments are coloured in black, blue, and red, respectively).

The inspection of the results from the design also highlighted an important limitation of RENATE. The products generated from Talazoparib contain shuffled key fragments compared to those in its reference ligand. This is due to the heuristics applied by the algorithm and the use of fingerprint‐based scoring, which sometimes cannot match the global shape and features of candidates with their references. Most of these molecules were filtered out by the scoring functions but some of them can still describe valid interactions by chance. These compounds might still be of interest, but they were not produced using a rational approach. A high number of these products was found for Talazoparib due to its connections and complexity, which as previously discussed, limited the number of structures generated by the algorithm, hence reducing the chance of finding better solutions. Examples of valid and invalid candidates from Talazoparib are reported in Figure 6. Talazoparib can be seen as a three‐fragment molecule (B−A−C): The main interacting scaffold (A) plus two substituents (B and C, five‐ and six‐membered rings, respectively), which are directly connected to the scaffold. Although Row2 and Row606 are both predicted to be active by the QSAR model, they are considered valid and invalid candidates, respectively, since the first has a configuration identical to the query (B−A−C), while the second has a different configuration (A−B−C).

### Compound synthesis

**Figure 7:**
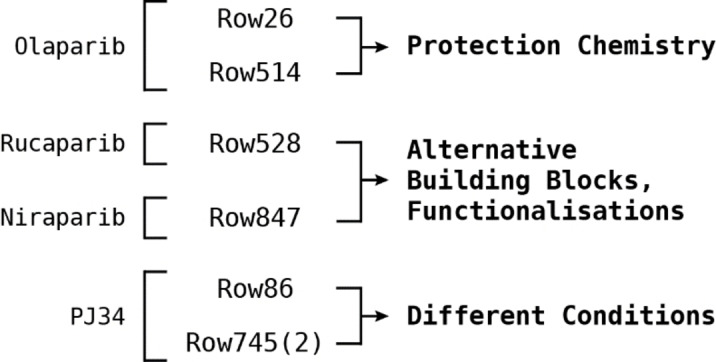
Synthesis summary. The diagram describes the names of the reference ligands (e. g. Olaparib) on the left, which are connected to their candidate structures (e. g. Row26). Candidates are associated with descriptions on the right side, which refer to additional/alternative chemistry (e. g. protection chemistry).

The synthetic routes proposed by RENATE for the selected candidates were adjusted according to three factors: reagent availability (e. g. cost of building blocks), additional steps (e. g. protection chemistry), and reaction conditions (e. g. yields, catalysts, solvents) leading to the selection of six candidates which are reported in Table 2 (Row26, Row86, Row514, Row847, Row528, Row745(2)). Table 2 also includes two intermediates of the selected compounds (Row86(I) and Row 528(I)). Proposed and adjusted routes are reported in the Supporting Information (Section S6), where the additional steps are highlighted in dashed squares. The outcomes of the syntheses are reported in Figure 7. Compound purities, and numbers of proposed and actual synthetic steps are reported in Table 2.

**Table 2:** Results from the synthesis of the selected candidates, where purities are reported along with the numbers of proposed and actual synthetic steps needed to obtain the compounds. (a) Intermediate compound. (b) (1S,2S) isomer of Row745, which was selected over the other stereo‐enumerated compounds from the docking. (c) These compounds were selected from the docking with a specific stereo configuration, but their synthesis produced mixtures, hence their structures are represented as flattened.

Reference	Candidate	Structure	Purity	Proposed steps	Actual steps
Olaparib	Row26	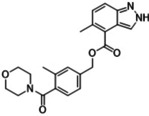	94 %	2	4
Olaparib	Row514	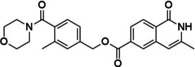	88 %	2	5
Rucaparib	Row528 (I)a	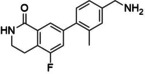	98 %	N.A.	N.A.
Rucaparib	Row528	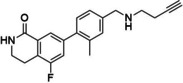	91 %	1	5
Niraparib	Row847	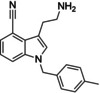	98 %	1	4
PJ34	Row86 (I)a	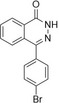	94 %	N.A.	N.A.
PJ34	Row745(2)b, c	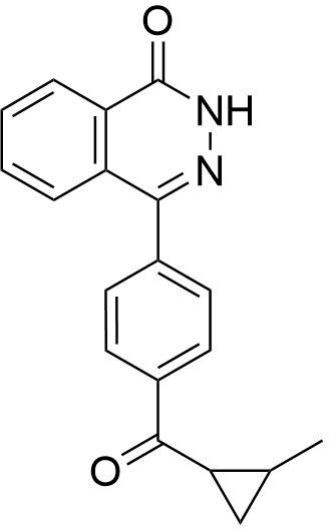	87 %	1	2
PJ34	Row86c	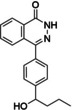	97 %	1	1

Row86 and Row745(2) (PJ34 candidates) were obtained via organolithium conditions rather than a Grignard generation since the latter was considered less robust; hence, these molecules were obtained through procedures very similar to their original routes. Note that Row745(2) and Row86 were produced as racemates of diastereomers or enantiomers. Other routes where minor adjustments were made are those of Row26 and Row514 (Olaparib candidates), where some protection chemistry was introduced since, as we discussed in our previous publications, the reaction vector approach does not account for the presence of reactive groups outside the reaction centre [20, 50].

More significant modifications were introduced in the synthesis of Row847 (Niraparib candidate), which was redesigned using a precursor of the building block proposed by the algorithm. As a consequence of the use of a precursor, the synthesis also required further functionalisation (i. e., extra steps) to obtain the final compound. A similar process is described for Row528 (Rucaparib candidate) with the exception that the precursor also required a different reaction to form a C−C bond between the two aryl rings.

### PARP1 activity assay

The synthesised compounds (Row26, Row86, Row514, Row528, Row745(2), Row847) were assayed along with two intermediates (Row528 (I) and Row86 (I)) for their inhibitory activity against PARP1 (Table 3). Among the assayed compounds, the PJ34 candidates (Row86 (I), Row86, and Row745(2)) reported potencies in the sub‐micromolar range, with the intermediate Row86 (I) emerging as the most potent inhibitor with IC_50_=395 nM. These molecules share the same phthalazinone ring system, which although not innovative within the PARP literature, was not present in the reference ligand PJ34 used as an input to the software. Additional active compounds are Row514 (derived from Olaparib) which exhibited IC_50_=19 μM, and Row847 (derived from Niraparib) showing IC_50_=16 μM. It is worth noting that Row847 does not contain the typical benzamide moiety which is involved in the key interactions with the catalytic domain of PARP1s, and characterises most of the PARP1 inhibitors which work as nicotinamide mimetics. This result may suggest this novel chemotype as a starting point for the potential identification of new inhibitors.

**Table 3:** PARP1 inhibitory activities and standard deviations. Activities were averaged on three replicas for every compound with potent inhibition. Compounds with IC50 above 1 μM were measured once.

Compound	IC_50_ (pIC_50_±SEM)
Row26	No inhibition
Row514	19.0 μM
Row528 (I)	No inhibition
Row528	No inhibition
Row847	16.0 μM
Row86 (I)	395 nM (6.40±0.096)
Row745(2)	908 nM (6.04±0.039)
Row86	755 nM (6.12±0.300)

### PAMPA permeability

Based on the results from the PARP1 activity assay, two compounds were preliminary evaluated for their BBB permeability using PAMPA. In particular, we selected Row514 (derived by Olaparib) and one of the phthalazinone derivatives, Row745(2) (derived by PJ34). Olaparib was also included as reference compound. Results are reported in Table 4, which show that Row745(2) has good BBB permeability with a diffusion of 2.1×10^−6^ cm s^−1^. A lower permeability is shown for Row514; however, this is approximately 20 times greater than that of its reference Olaparib. This demonstrates that the design strategy was successful in identifying a micromolar compound with improved brain penetration in a single design cycle.

**Table 4:** Apparent BBB permeability (P_app_) and membrane retention (MR) measures obtained from the PAMPA.

Compound	PAMPA BBB P_app_ (10^−6^ cm s^−1^)	MR (%)
Olaparib	0.016	3.0
Row514	0.325	0.8
Row745(2)	2.100	3.6

## CONCLUSIONS AND FUTURE OUTLOOK

We have described the application of reaction vectors to the design and synthesis of novel and synthetically accessible PARP1 inhibitors with improved BBB penetration, compared to the reference compounds on which the designs are based. Our approach involved the use of data from known PARP1 inhibitors and their crystal structures, in combination with docking, machine learning, and our reaction vector‐based tool RENATE. We were able to use RENATE to design compounds that were predicted to meet the multi‐objectives of the study and which have high structural diversity to their reference ligands. The software also proposed viable synthetic routes that allowed the preparation of selected compounds which were biologically assessed against PARP1 with resulting activities in the order of micromolar concentration (IC_50_ values ranging from 0.4 to 19 μM). Most of the compounds share the benzamide moiety that typically characterises the inhibitors of PARP1; however, compound Row847 emerged as an innovative hit among these. Although the indole scaffold imparted a weak activity to the compound (IC_50_=16 μM), its novelty provides promising insights for the development of new series of PARP1 inhibitors. We also experimentally validated the brain penetration of two compounds that showed binding with PARP1. The results obtained from the permeability measurements showed that RENATE was able to account for the optimisation of the brain penetration of compounds within just one design cycle, hence promoting its suitability for generating valid alternatives to known compounds. We conclude by suggesting that this work constitutes the first example in the literature of a *de novo* design method where, as well as successfully designing novel hit compounds with desired pharmaceutical properties, our software also provided multi‐step synthetic routes which led to the preparation of the compounds in the laboratory.

## EXPERIMENTAL SECTION

### Computational methods

A summary of the theory and implementation of reaction vectors for *de novo* design is reported at the end of this section along with references that provide detailed explanations.

**Figure 8:**
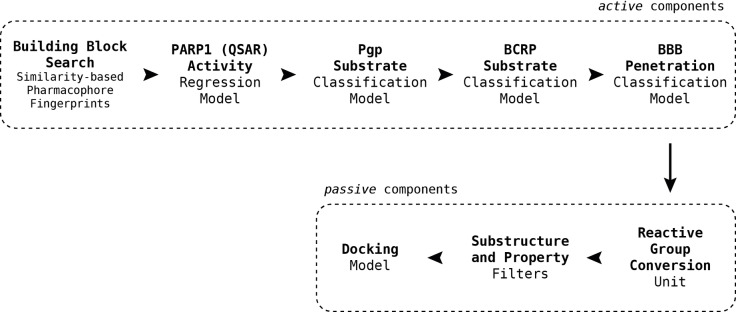
RENATE scoring module for the PARP1 inhibitors design. The active components drive the algorithm at each step of the design, while the passive components are applied at the end to refine the selection of the most promising candidates.

The scoring module implemented for the PARP1 design is described in Figure 8, and is divided into five active components applied sequentially at each step of the design, and three passive components applied at the end of the design. The active components consist of a similarity search to retrieve building blocks similar to the fragments extracted from the reference ligands, and four machine learning models to score the structures generated by RENATE. The models consist of a PARP1 activity regression model, and Pgp substrate, BCRP substrate, and BBB penetration classification models. The passive components consist of a reactive group conversion unit, substructure and property filters, and a docking model. Each component and the data used to run the experiment are discussed in the following sections.

#### Reagent and reaction data and building block scoring

The 746,245 reagent set from Enamine and the 92,530 USPD reaction vector database described in Ghiandoni et al. [50] were selected as sources of reagent and reaction data for RENATE, respectively. Count FeatMorgan fingerprints (Radius 2) 1024‐bit and Euclidean distance were selected for the scoring of building blocks. FeatMorgan fingerprints are described in the Supporting Information (Section S1). The use of pharmacophore fingerprints aims to maximise the chance of retrieving isosteric replacements of the query fragments.

#### Machine learning models

A PARP1 activity data set of 2,371 entries was obtained from ChEMBL 24 in January 2019 [51]. The Pgp data was obtained from a collection of annotated substrates/non‐substrates of Pgp from the literature [52]. The BCRP data was also obtained from a set of substrates/non‐substrates of BCRP from the literature [53]. The BBB data was obtained from AdmetSAR [54]. Only entries associated with defined units and activities were retained. Activities were converted into micromolar pIC_50_ values. Molecules were sanitised, salts and ions were stripped, and canonical SMILES were generated using RDKit [55]. SMILES associated with multiple activities were grouped and values were averaged.

The standardised PARP1 (1363 actives, 501 inactives based on an activity threshold of 1 μM), Pgp (243 substrates, 241 non‐substrates), BCRP (164 substrates, 99 non‐substrates), and BBB (1,437 BBB+, 401 BBB−) data sets were described using a selection of fingerprints and descriptors using RDKit, which were used to train a series of Random Forest models [55, 56]. The models were evaluated by performing an internal validation using 80 % train and 20 % test data. The PARP1 models were evaluated on their R^2^ score, mean absolute error (MAE), and mean squared error (MSE). The Pgp, BCRP, and BBB models were evaluated on Recall, Precision, F1‐score, and Matthews correlation coefficient (MCC) metrics weighted by class sample size calculated using Scikit‐learn [56]. The validation was repeated 15 times per model using random train‐test splits. The best performing models were optimised on their hyper‐parameters via 5‐fold cross‐validation and a genetic algorithm to yield the models used in the design workflow. In addition, the Pgp and BCRP models were optimised on their descriptors via feature elimination. The descriptors and performance metrics of the optimised models are reported in Table 5. The other models are described in the Supporting Information (Section S1).

**Table 5:** The optimised models for PARP1 activity regression and Pgp substrate, BCRP substrate, and BBB penetration classification. The models are described on their molecular descriptors and performance metrics. The implementations of the selected molecular descriptors are reported in the Supporting Information (Section S1).

Data set	Descriptors	Performance metrics
PARP1 activity	Count Morgan (Radius 2)–1024 bits	R^2^ 0.79, MAE 0.41, MSE 0.31
Pgp substrate	2D (Atom/Bond Counts, BCUT, Chi and Kappa, GCUT, SlogP, SMR, VSA)–44 features	Recall 0.80, Precision 0.81, F1‐score 0.80, MCC 0.62
BCRP substrate	2D (Atom/Bond Counts, BCUT, Chi and Kappa, GCUT, SlogP, SMR, VSA)–103 features	Recall 0.79, Precision 0.80, F1‐score 0.78, MCC 0.55
BBB penetration	Count Morgan (Radius 2)–1024 bits	Recall 0.92, Precision 0.91, F1‐score 0.91, MCC 0.75

#### Docking model

A PARP1 docking model was validated by cross‐docking the reference ligands to the crystal structure of Niraparib (PDB ID: 4R6E) (which is the one with the highest resolution) using GOLD [57]. Waters were extracted, and PLP and GoldScore functions were selected for pose and interaction scoring. The software parameters are reported in the Supporting Information (Section S2). Prior to the validation, the reference ligands were prepared by sanitising them using RDKit and by calculating their protonation states at pH 7.4 using MOE [58]. In addition, their stereocentres were enumerated and conformations were minimised using the MMFF94 method.

**Figure 9:**
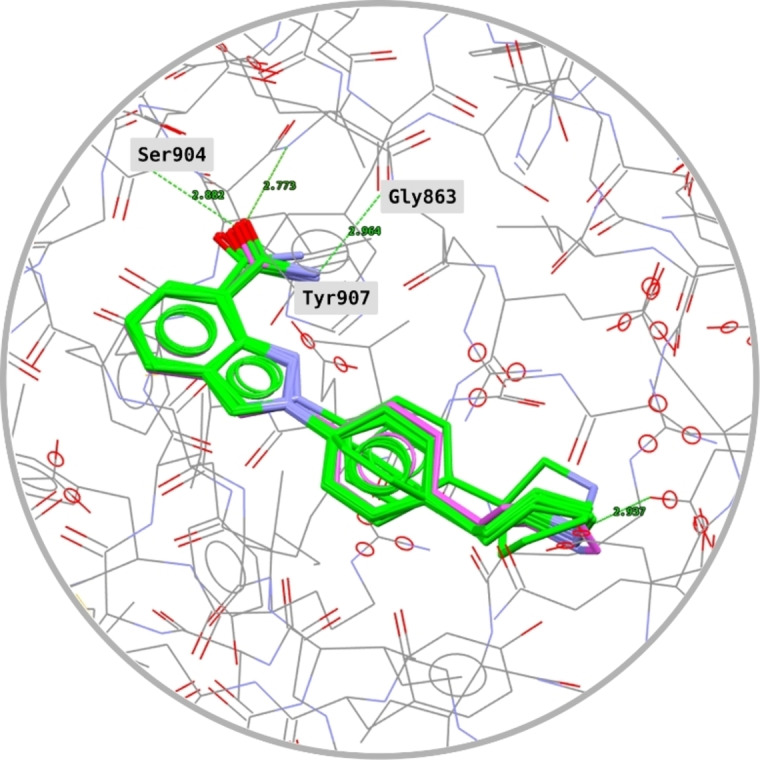
Overlap between docked (green) and co‐crystal (purple) poses of Niraparib. Ten computed poses out of ten produced good overlap with the experimental data.

**Figure 10:**
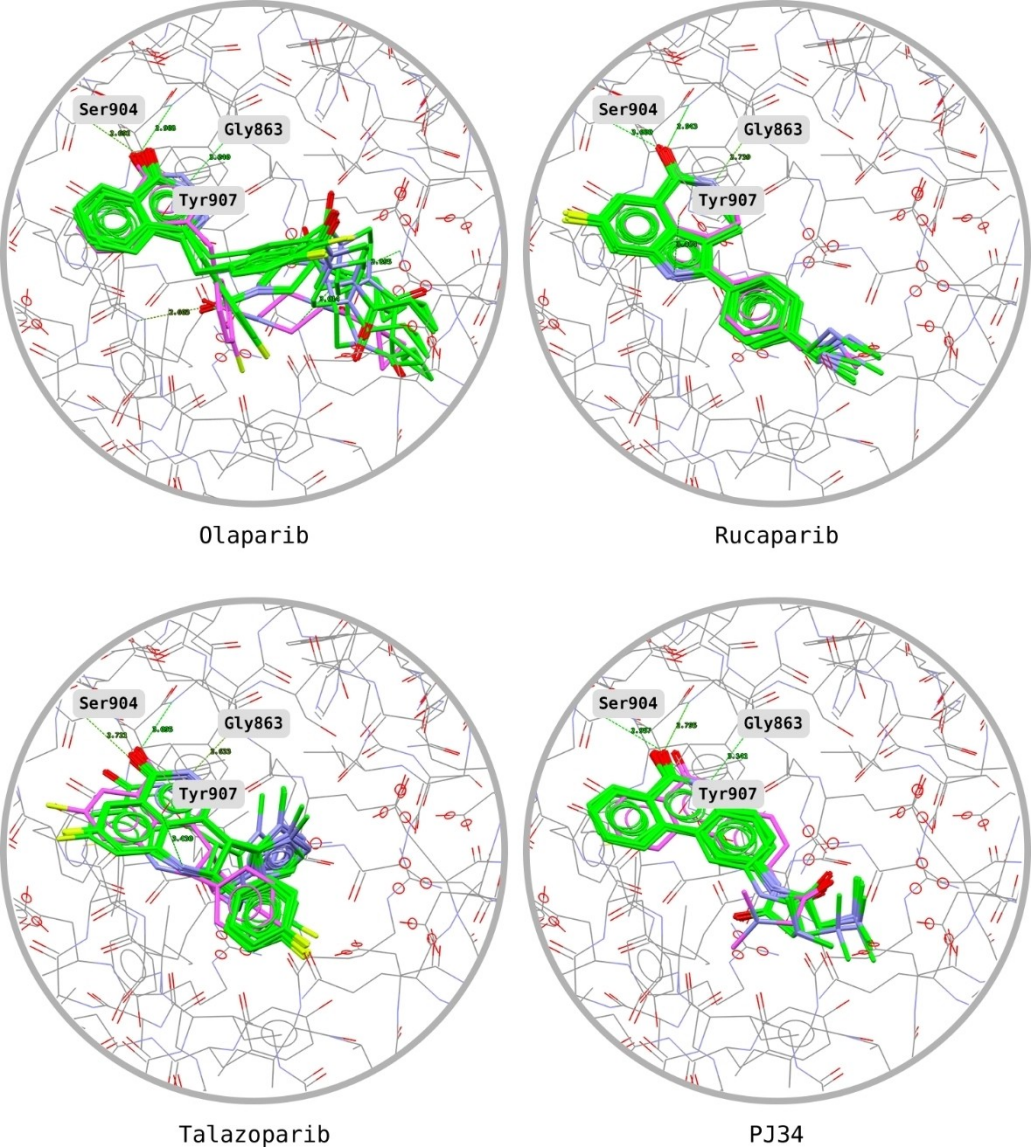
Overlap between docked (green) and experimental (purple) poses of Olaparib, Rucaparib, Talazoparib, and PJ34. The ligands are shown within the binding pocket whilst interacting with the key residues Ser904, Gly863, and Tyr907. Olaparib produced the largest pose variance although its portion binding with the key residues still produced good overlap with the experimental pose.

The model was validated by docking the reference ligands plus their virtually generated stereoisomers: Each ligand generated 10 poses, which were compared with those from the co‐crystals. The superimpositions between computed and co‐crystal poses of Niraparib are described in Figure 9, while those of the other ligands are in Figure 10. Mean PLP.Fitness and GoldScore.Fitness and the number of consistent poses (i. e., correct overlap with the co‐crystal) are reported in the Supporting Information (Section S2). Note that the virtually generated stereoisomers, which represented structures almost identical to those of the actual inhibitors, always produced lower PLP.Fitness scores. This result suggests that the parametrisation selected for the docking can discriminate effectively also on the stereochemistry of compounds.

#### Reactive group conversion and additional filters

The reactive group conversion consisted of a SMARTS filter to detect reactive compounds followed by a reaction vector structure generation step consisting of functional transformations only in order to convert the reactive groups into non‐reactive functional groups. After the conversion, compounds were reprocessed through the filter and those still containing reactive patterns were discarded. The filter was implemented using the definitions proposed by Hann and colleagues and RDKit [59]. Note that compounds modified by the reactive group conversion were also rescored by the models.

The substructure and property filters were configured to remove compounds matching any pattern from a selection of SMARTS and those violating more than one Lipinski rule. The SMARTS were implemented using RDKit from the definitions in Brenk et al. [60], Doveston et al. [61], Baell and Holloway [62], and ZINC (http://blaster.docking.org/filtering/).

#### Reaction vectors and their application in de novo design

A summary of reaction vectors and the associated structure generation algorithm, which enables them to be applied in de novo design, is given below. More details are provided in Patel et al. [18], Hristozov et al. [19], Gillet et al. [63], and several works by Ghiandoni et al. [20, 50, 64].

Reaction vectors encode the structural changes that occur in a chemical reaction as difference vectors, as shown in Equation (1). The reaction components are described using atom pair descriptors (AP) which describe two atoms and their properties, along with a separator that indicates the length of the atom path between the two atoms. Two types of AP descriptors are used in the reaction vector, namely AP2s which describe neighbouring atoms with the separator encoding information about the bond type, and AP3s which describe a pair of atoms separated by two bonds. An AP2 is represented as shown in Equation 2.

(1) ReactionVector=[∑ProductVectors]-[∑ReactantVectors]

Equation (1): Generic definition of a reaction vector.

(2) $$X_{1}\left(h_{1}p_{1}r_{1}\right)-S\left(BO\right)-X_{2}\left(h_{2}p_{2}r_{2}\right)$$

Equation (2): AP2 descriptor.

In AP2s, $X_{1}$ and $X_{2}$ are the atom types; $h_{1}$ and $h_{2}$ are the numbers of non‐hydrogen bonds incident on each atom; $p_{1}$ and $p_{2}$ are the numbers of π electrons shared by the respective atom; $r_{1}$ and $r_{2}\;$ are the numbers of rings each atom is part of; S is the separator; BO is the connection bond order which can be 1 (single), 2 (double), 3 (triple) or 4 (aromatic). AP3s describe the two atoms at the start and end of the path only, i. e., there is no bond information. The atom pair vectors are counts indicating the number of times each atom pair occurs in a reaction component. Reaction vectors are generated by first cleaning a reaction to ensure it is balanced, i. e., it contains the same number of heavy atoms on each side of the reaction. Then AP2 and AP3 descriptors are calculated for each component and are summed for the reactants and the products, respectively. Finally, the reactant descriptors are subtracted from the product descriptors so that the reaction vector encodes the atom pairs that are changed in the reaction. The reaction vector consists of atom pairs with negative counts, which indicate atom pairs lost during the reaction, and positive atom pairs which indicate atom pairs gained during the reaction.

The structure generation process consists of applying a reaction vector to a new reactant(s). The first step is to test for validity. A reaction vector is considered valid if the reactant contains (either wholly or partially) the negative atom pairs encoded in the reaction vector. If the match is partial, then it is necessary to identify a reagent that contains the missing negative atom pairs. The negative atom pairs are then used to fragment the reactants and the products are assembled by adding in atoms according to the positive atom pairs. In the first implementation of the structure generation algorithm, described in Patel et al. [19] the structure generation proceeded atom‐by‐atom in a breadth first search with back‐tracking. Although this was an effective approach to generate novel molecules, it was slow in execution. A considerably faster implementation has since been developed in which fragmentation and recombination paths (or fragments) are stored with the reaction vector, as described in Ghiandoni et al. [64].

### Chemistry methods

All reactions were carried out using anhydrous organic solvents under a nitrogen atmosphere at room temperature unless otherwise stated. All solvents, reagents and catalysts were obtained from commercial sources and were used without further purification unless otherwise stated. All reactions were carried out using oven‐dried glassware. All microwave reactions were carried out in a Biotage® Initiator+ using a maximum power of 400 W. Reactions were monitored using TLC and/or LCMS. TLC was performed using glass pre‐coated silica gel plates and visualized using either ultraviolet light (254 nm) or by dipping in potassium permanganate or phosphomolybdic acid solution and heating. Flash column chromatography was performed using a Biotage® Isolera 4 using pre‐packed Biotage® SNAP KP‐Sil cartridges (40–63 μm) unless otherwise noted. Preparative HPLC was performed in reverse phase using a Waters XBridgeTM C18 column (30 mm×100 mm, 5 μm) at room temperature using an injection volume of 1500 μL at a flow rate of 40 mL min^−1^ at 10 % B for 2.00 min then a gradient of 10–95 % B over 14.00 min and held for 2.00 min, where A=0.2 % ammonium hydroxide in water and B=0.2 % ammonium hydroxide in acetonitrile. 1H NMR spectra were recorded in chloroform‐d, DMSO‐d6 or methanol‐d4 at 400 or 500 MHz. Chemical shifts are reported in ppm with reference to the residual solvent peak. Multiplicities are reported with coupling constants (J) in hertz (Hz) and are given to the nearest 0.1 Hz. The peak information is described as: s=singlet, d=doublet, t=triplet, q=quartet, m=multiplet, br=broad. Analytical HPLC and LCMS analyses were performed using seven methods: Method A (Kinetex Core shell C18 column (2.1 mm×50 mm, 5 μm; temperature: 40 °C), injection volume 3 μL, flow rate 1.2 mL min^−1^, 0.1 % formic acid in H_2_O/0.1 % formic acid in MeCN; 0–1.20 min gradual change from 5 to 100 % MeCN; 1.20–1.30 min 100 % MeCN; 1.30–1.31 min gradual change to 5 % MeCN; 1.31–1.70 min 5 % MeCN. UV spectra recorded at 215 nm, spectrum range 210—420 nm; mass spectra obtained using a 2010EV or Waters ZQ detector; ionization mode: electrospray positive), Method B (Phenomenex Kinetex‐XB C18 column (2.1 mm×100 mm, 1.7 μm; temperature: 40 °C), injection volume 1 μL, flow rate 0.6 mL min^−1^, 0.1 % formic acid in H_2_O/0.1 % formic acid in MeCN; 0–5.30 min gradual change from 5 to 100 % MeCN; 5.30–5.80 min 100 % MeCN; 5.80–5.82 min gradual change to 5 % MeCN; 5.82–7.00 min 5 % MeCN. UV spectra recorded at 215 nm, spectrum range 200—400 nm; mass spectra obtained using a Waters SQD, SQD2 or QDA detector; ionization mode: electrospray positive), Method C (Waters UPLCT™ BEH™ C18 column (2.1 mm×50 mm, 1.7 μm; temperature: 40 °C), injection volume 1 μL, flow rate 0.9 mL min^−1^, 0.1 % formic acid in H_2_O/0.1 % formic acid in MeCN; 0–1.10 min gradual change from 5 to 100 % MeCN; 1.10–1.35 min 100 % MeCN; 1.35–1.40 gradual change to 5 % MeCN; 1.40–1.50 min 5 % MeCN. UV spectra recorded at 215 nm, spectrum range 200–400 nm; mass spectra obtained using a Waters SQD, SQD2 or QDA detector; ionization mode: electrospray positive), Method D (Waters UPLC™ BEH™ C18 column (2.1 mm×30 mm, 1.7 μm; temperature: 40 °C), injection volume 1 μL, flow rate 1.0 mL min^−1^, 2 mM ammonium bicarbonate in water/MeCN; 0–0.75 min gradual change from 5 to 100 % MeCN; 0.75–0.85 min 100 % MeCN; 0.85–0.90 gradual change to 5 % MeCN; 0.90–1.10 min 5 % MeCN. UV spectra recorded at 215 nm, spectrum range 200—400 nm; mass spectra obtained using a Waters Quattro Premier XE detector; ionization mode: electrospray positive), Method E (Waters UPLC™ BEH™ C18 column (2.1 mm×100 mm, 1.7 μm; temperature: 40 °C), injection volume 1 μL, flow rate 0.6 mL min^−1^, 2 mM ammonium bicarbonate in water/MeCN; 0–5.30 min gradual change from 5 to 100 % MeCN; 5.30–5.80 min 100 % MeCN; 5.80–5.82 min gradual change to 5 % MeCN; 5.82–7.00 min 5 % MeCN. UV spectra recorded at 215 nm, spectrum range 200—400 nm; mass spectra obtained using a Waters Quattro Premier XE or SQD2 detector; ionization mode: electrospray positive), Method F (Waters Atlantis™ dC18 column (2.1 mm×100 mm, 3 μm; temperature: 40 °C), injection volume 3 μL, flow rate 0.6 mL min^−1^, 0.1 % formic acid in H_2_O/0.1 % formic acid in MeCN; 0–5.00 min gradual change from 5 to 100 % MeCN; 5.00–5.40 min 100 % MeCN; 5.40–5.42 min gradual change to 5 % MeCN; 5.42–7.00 min 5 % MeCN. UV spectra recorded at 215 nm, spectrum range 210—420 nm; mass spectra obtained using a 2010EV detector; ionization mode: electrospray positive), or Method G (Phenomenex Gemini NX C18 column (2.0 mm×100 mm, 3 μm; temperature: 40 °C), injection volume 3 μL, flow rate 0.6 mL min^−1^, 2 mM ammonium bicarbonate in water/MeCN; 0–5.50 min gradual change from 5 to 100 % MeCN; 5.50–5.90 min 100 % MeCN; 5.90–5.92 min gradual change to 5 % MeCN; 5.92–7.00 min 5 % MeCN. UV spectra were recorded at 215 nm, spectrum range 200–420 nm; mass spectra were obtained using a Waters ZQ detector; ionization mode: electrospray positive). All reported final compounds were analyzed with one of these analytical methods with purities >98 % unless otherwise stated.

#### Synthesis of compounds Row26 and Row514

**[(4‐Bromo‐3‐methylphenyl)methoxy](tert‐butyl)dimethylsilane (1)**

A solution of (4‐bromo‐3‐methyl‐phenyl)methanol (500 mg, 2.49 mmol), imidazole (203 mg, 2.98 mmol) and tert‐butyl‐chloro‐dimethyl‐silane (450 mg, 2.98 mmol) in DMF (8 mL) was stirred 18 h at ambient temperature. The reaction was then partitioned between Et_2_O (40 mL) and water (30 mL). The aqueous layer was extracted once more with Et_2_O (40 mL) and the combined organic layers dried (MgSO_4_) and concentrated *in vacuo*. Purification by flash chromatography using 0–100 % tert‐butyl methyl ether in heptane gave the title compound as a yellow oil of 80 % purity (730 mg, 1.85 mmol, 74 %). ^1^H NMR (400 MHz, Chloroform‐d) δ=7.47 (d, *J=*8.1 Hz, 1H), 7.18 (s, 1H), 7.00 (d, *J=*7.8 Hz, 1H), 4.65 (s, 2H), 2.39 (s, 3H), 0.94 (s, 9H), 0.09 (s, 6H). LCMS (Method C) RT 0.95 min, no mass ion detected.

**4‐(4‐{[(tert‐Butyldimethylsilyl)oxy]methyl}‐2‐methylbenzoyl)morpholine (2)**

To a cooled (−78 °C) solution of **1** (837 mg, 2.12 mmol, 80 % purity) in THF (13.4 mL) was added a solution of morpholine‐4‐carbonyl chloride (0.37 mL, 3.19 mmol) dropwise. The reaction was stirred for 1 h at −78 °C then *n‐*butyllithium (1.6 mL, 2.34 mmol, 1.47 M) was added dropwise. The reaction was stirred for 30 min at −78 °C then allowed to warm to 0 °C over 3 h. The solution was diluted with saturated aqueous NaHCO_3_ (5 mL) then partitioned between EtOAc (20 mL) and water (20 mL). The aqueous layer was extracted once more with EtOAc (20 mL) and the combined organic layers dried (MgSO_4_) and concentrated *in vacuo*. Purification by flash chromatography using 0–60 % EtOAc in heptane gave the title compound as a yellow gum (292 mg, 0.79 mmol, 37 %). ^1^H NMR (500 MHz, Chloroform‐d) δ=7.11–6.88 (m, 3H), 4.60 (s, 2H), 3.84–2.95 (m, 8H), 2.21 (s, 3H), 0.84 (s, 9H), 0.00 (s, 6H). LCMS (Method C) RT 1.16 min, *m/z=*350 [M+H]^+^.

**[3‐Methyl‐4‐(morpholine‐4‐carbonyl)phenyl]methanol (3)**

Tetrabutylammonium fluoride (2.3 mL, 2.30 mmol, 1 M solution in THF) was added to a solution of **2** (277 mg, 0.75 mmol) in THF (2.4 mL) and the reaction stirred 1 h at ambient temperature then concentrated *in vacuo*. Purification by flash chromatography using 0–10 % MeOH in DCM gave the title compound as a yellow gum of 90 % purity (257 mg, 0.98 mmol, >100 %). ^1^H NMR (400 MHz, Chloroform‐d) δ=7.23–7.19 (m, 2H), 7.15 (d, *J=*7.7 Hz, 1H), 4.67 (s, 2H), 3.76–3.63 (m, 4H), 3.60–3.48 (m, 2H), 3.33–3.14 (m, 2H), 2.32 (s, 3H). The OH proton was not observed. LCMS (Method D) RT 0.32 min, *m/z=*236 [M+H]^+^.

**Methyl 3‐methyl‐1‐oxo‐1,2‐dihydroisoquinoline‐6‐carboxylate (4)**

COware® chamber A was charged with a solution of 6‐bromo‐3‐methyl‐2H‐isoquinolin‐1‐one (50 mg, 0.21 mmol), triethylamine (0.11 mL, 0.76 mmol) and [1,1′‐bis(diphenylphosphino)ferrocene]dichloropalladium(II) (7.7 mg, 0.011 mmol) in MeOH (1 mL) and DMF (1 mL) and degassed with nitrogen for 10 min. COware® chamber B was then charged with a solution of formic acid (24 μL, 0.63 mmol) in toluene (1 mL) to which was added methanesulfonyl chloride (49 μL, 0.63 mmol) and triethylamine (0.18 mL, 1.26 mmol) and the reaction heated at 75 °C for 18 h. After cooling, concentration *in vacuo* gave the title compound as a brown solid of 45 % purity (100 mg, 0.207 mmol, 99 %). ^1^H NMR (400 MHz, DMSO‐d6) δ=11.48 (s, 1H), 8.30–8.09 (m, 2H), 7.88 (dd, *J=*8.3, 1.6 Hz, 1H), 6.49 (s, 1H), 3.91 (s, 3H), 2.23 (s, 3H). LCMS (Method D) RT 0.44 min, *m/z=*218 [M+H]^+^.

**3‐Methyl‐1‐oxo‐1,2‐dihydroisoquinoline‐6‐carboxylic acid (5)**

Lithium hydroxide (16 mg, 0.62 mmol) was added to a solution of **4** (100 mg, 0.207 mmol, 45 % purity) in THF (0.5 mL) and water (0.5 mL) and the reaction stirred 16 h at ambient temperature. Further lithium hydroxide (16 mg, 0.62 mmol) was added and the reaction heated at 50 °C for 2 h. Lithium hydroxide (16 mg, 0.62 mmol) was added and the reaction heated at 50 °C for 16 h. After cooling, the reaction was partitioned between EtOAc (5 mL) and water (5 mL). The aqueous layer was isolated and 2 M aqueous HCl added until pH 3 had been attained. The aqueous layer was then extracted with EtOAc (4×) and these combined organic layers dried (MgSO_4_) and concentrated *in vacuo* to give the title compound as a yellow solid of 75 % purity (45 mg, 0.166 mmol, 80 %). ^1^H NMR (400 MHz, DMSO‐d6) δ=11.43 (s, 1H), 8.19 (d, *J=*8.3 Hz, 1H), 8.12 (d, *J=*1.3 Hz, 1H), 7.86 (dd, *J=*8.3, 1.6 Hz, 1H), 6.46 (s, 1H), 2.22 (s, 3H). The carboxylic acid proton was not observed. LCMS (Method A) RT 0.86 min, *m/z=*204 [M+H]^+^.

**[3‐Methyl‐4‐(morpholine‐4‐carbonyl)phenyl]methyl 3‐methyl‐1‐oxo‐1,2‐dihydroisoquinoline‐6‐carboxylate (Row514)**

A solution of **5** (45 mg, 0.166 mmol, 75 % purity), **3** (67 mg, 0.256 mmol, 90 % purity) and triphenylphosphine (74 mg, 0.284 mmol) in THF (3 mL) cooled to 5 °C was treated with diisopropyl azodicarboxylate (56 μL, 0.284 mmol). The reaction was then stirred for 16 h at ambient temperature then partitioned between EtOAc (5 mL) and water (5 mL). The aqueous layer was extracted with EtOAc (2×) and the combined organic layers dried (MgSO_4_) and concentrated *in vacuo*. Purification by preparative HPLC followed by flash chromatography using 40–100 % EtOAc in heptanes followed by 0–10 % MeOH in EtOAc gave the title compound as a white solid of 88 % purity (8.2 mg, 0.017 mmol, 10 %). ^1^H NMR (500 MHz, Chloroform‐d) δ=9.38 (s, 1H), 8.40 (d, *J=*8.4 Hz, 1H), 8.18 (d, *J=*1.4 Hz, 1H), 8.03 (dd, *J=*8.4, 1.6 Hz, 1H), 7.35–7.29 (m, 1H), 7.26–7.19 (m, 2H), 6.36 (s, 1H), 5.37 (s, 2H), 3.86–3.80 (m, 2H), 3.80–3.75 (m, 2H), 3.62–3.54 (m, 2H), 3.29–3.21 (m, 2H), 2.38–2.36 (m, 3H), 2.35 (s, 3H). LCMS (Method E) RT 2.58 min, *m/z=*421 [M+H]^+^.

**[3‐Methyl‐4‐(morpholine‐4‐carbonyl)phenyl]methyl 5‐methyl‐1H‐indazole‐4‐carboxylate (Row26)**

The title compound was prepared in an analogous manner to compound **Row514** using **3**, 5‐methyl‐1H‐indazole‐4‐carboxylic acid and diethyl azodicarboxylate. Purification was performed by reverse phase chromatography using 30–95 % MeCN in water with 0.2 %% ammonium hydroxide. ^1^H NMR (400 MHz, Methanol‐d4) δ=8.19 (s, 1H), 7.67–7.62 (m, 1H), 7.51–7.41 (m, 2H), 7.34 (d, *J=*8.2 Hz, 1H), 7.27 (d, *J=*8.2 Hz, 1H), 5.47 (s, 2H), 3.85–3.69 (m, 4H), 3.66–3.51 (m, 2H), 3.34–3.21 (m, 2H), 2.67 (s, 3H), 2.34 (s, 3H). LCMS (Method E) RT 2.58 min, *m/z=*394 [M+H]^+^.

#### Synthesis of compounds Row528 and Row528 (I)

**5‐Fluoro‐7‐(4,4,5,5‐tetramethyl‐1,3,2‐dioxaborolan‐2‐yl)‐1,2,3,4‐tetrahydroisoquinolin‐1‐one (6)**

A suspension of 7‐bromo‐5‐fluoro‐1,2,3,4‐tetrahydroisoquinolin‐1‐one (400 mg, 1.64 mmol), bis(pinacolato)diboron (624 mg, 2.46 mmol), 2‐(dicyclohexylphosphanyl)‐2′,4′,6′‐tris(isopropyl)biphenyl (78 mg, 0.164 mmol) and potassium acetate (488 mg, 4.92 mmol) in 1,4‐dioxane (15 mL) was degassed with nitrogen and tris(dibenzylideneacetone)dipalladium(0) (75 mg, 0.082 mmol) added. The mixture was irradiated to 80 °C in a microwave reactor for 16 h. After cooling, the reaction mixture was diluted with water (15 mL) and extracted with EtOAc (3x). The combined organic layers were washed with brine (25 mL), dried (MgSO_4_) and concentrated *in vacuo*. Purification by flash chromatography using 0–100 % EtOAc in heptane followed by 0–10 % MeOH in EtOAc gave the title compound as a tan solid of 89 % purity (400 mg, 1.22 mmol, 75 %). ^1^H NMR (500 MHz, DMSO‐d6) δ=8.06 (s, 1H), 8.05–8.01 (m, 1H), 7.52–7.44 (m, 1H), 3.40 (td, *J=*6.6, 2.8 Hz, 2H), 2.92 (t, *J=*6.6 Hz, 2H), 1.31 (s, 12H). LCMS (Method C) RT 0.90 min, *m/z=*292 [M+H]^+^.

**tert‐Butyl N‐[(4‐bromo‐3‐methylphenyl)methyl]carbamate (7)**

A suspension of 4‐bromo‐3‐methylbenzylamine hydrochloride (300 mg, 1.27 mmol) in THF (10 mL) was treated with tert‐butoxycarbonyl tert‐butyl carbonate (321 μL, 1.40 mmol) followed by *N*‐ethyl‐*N*‐isopropyl‐propan‐2‐amine (0.44 mL, 2.54 mmol). The reaction mixture was stirred at room temperature for 16 h then concentrated *in vacuo*. Purification by flash chromatography using 0–30 % EtOAc in heptane gave the title compound as a white solid (285 mg, 0.95 mmol, 75 %). ^1^H NMR (400 MHz, Chloroform‐d) δ=7.47 (d, *J=*8.1 Hz, 1H), 7.18–7.10 (m, 1H), 7.04–6.90 (m, 1H), 4.82 (s, 1H), 4.23 (d, *J=*5.7 Hz, 2H), 2.38 (s, 3H), 1.46 (s, 9H). LCMS (Method C) RT 1.09 min, *m/z=*244/246 [M−*t*‐Bu+H]^+^.

**tert‐Butyl N‐{[4‐(5‐fluoro‐1‐oxo‐1,2,3,4‐tetrahydroisoquinolin‐7‐yl)‐3‐methylphenyl]methyl}carbamate (8)**

A suspension of **7** (200 mg, 0.666 mmol), **6** (194 mg, 0.666 mmol) and potassium carbonate (373 mg, 2.70 mmol) in 1,4‐dioxane (3 mL) and water (1 mL) was degassed with nitrogen and tetrakis(triphenylphosphine)palladium(0) (38 mg, 0.033 mmol) added. The mixture was irradiated to 100 °C in a microwave reactor for 2 h, then at 120 °C for a further hour. After cooling, the reaction mixture was diluted with water (5 mL) and extracted with EtOAc (3×). The combined organic layers were washed with brine (10 mL), dried (MgSO_4_) and concentrated *in vacuo*. Purification by flash chromatography using 0–100 % EtOAc in heptane gave the title compound in two batches, a white solid of 89 % purity (123 mg, 0.28 mmol, 43 %) and a white solid of 80 % purity (100 mg, 0.23 mmol, 34 %). ^1^H NMR (400 MHz, Chloroform‐d) δ=7.78 (d, *J=*1.5 Hz, 1H), 7.15–7.02 (m, 4H), 6.21 (s, 1H), 4.81 (br s, 1H), 4.25 (d, *J=*5.5 Hz, 2H), 3.55 (td, *J=*6.7, 2.9 Hz, 2H), 2.99 (t, *J=*6.7 Hz, 2H), 1.41 (s, 9H), 2.20 (s, 3H). LCMS (Method C) RT 0.96 min, *m/z=*329 [M−*t*‐Bu+H]^+^.

**7‐[4‐(Aminomethyl)‐2‐methylphenyl]‐5‐fluoro‐1,2,3,4‐tetrahydroisoquinolin‐1‐one hydrochloride (Row528 (I))**

A solution of **8** (115 mg, 0.27 mmol, 89 % purity) in 1,4‐dioxane (1 mL) was treated with a solution of hydrogen chloride in 1,4‐dioxane (1.0 mL, 4.00 mmol, 4 M) and stirred at ambient temperature for 1 h. Water (200 μL) was added and the reaction stirred a further 10 min before concentrating *in vacuo*. The residue was suspended in 1,4‐dioxane (2 mL) and the solids isolated by filtration to give the title compound as a white solid (74.6 mg, 0.23 mmol, 85 %). ^1^H NMR (400 MHz, DMSO‐d6) δ=8.26 (br s, 3H), 8.14 (s, 1H), 7.62 (d, *J=*1.6 Hz, 1H), 7.44 (s, 1H), 7.43–7.35 (m, 2H), 7.30 (d, *J=*7.8 Hz, 1H), 4.04 (s, 2H), 3.45 (td, *J=*6.7, 2.8 Hz, 2H), 2.95 (t, *J=*6.6 Hz, 2H), 2.27 (s, 3H). LCMS (Method A) RT 0.56 min, *m/z=*285 [M+H]^+^.

**7‐(4‐{[(But‐3‐yn‐1‐yl)amino]methyl}‐2‐methylphenyl)‐5‐fluoro‐1,2,3,4‐tetrahydroisoquinolin‐1‐one (Row528)**

To a solution of but‐3‐ynyl methanesulfonate (10 μL, 0.125 mmol) and **Row528(I)** (40 mg, 0.125 mmol) in DMSO (1 mL) was added *N,N*‐diisopropylethylamine (44 μL, 0.249 mmol) and sodium iodide (1.9 mg, 0.013 mmol). The reaction mixture was warmed to 50 °C and stirred 5 h, then warmed to 60 °C and stirred 16 h. After cooling, the mixture was purified by preparative HPLC to give the title compound as white solid being of 91 % purity (3.7 mg 0.010 mmol, 8 %). ^1^H NMR (500 MHz, Chloroform‐d) δ=7.80 (d, *J=*1.6 Hz, 1H), 7.17 (s, 1H), 7.15–7.08 (m, 3H), 6.07 (s, 1H), 3.76 (s, 2H), 3.55 (td, *J=*6.7, 2.9 Hz, 2H), 3.00 (t, *J=*6.6 Hz, 2H), 2.77 (t, *J=*6.6 Hz, 2H), 2.37 (td, *J=*6.6, 2.6 Hz, 2H), 2.21 (s, 3H), 1.94 (t, *J=*2.6 Hz, 1H). One NH proton was not observed. LCMS (Method E) RT 3.07 min, *m/z=*337 [M+H]^+^.

#### Synthesis of compound Row847

**3‐Formyl‐1‐[(4‐methylphenyl)methyl]‐1H‐indole‐4‐carbonitrile (13)**

To a solution of 3‐formyl‐1H‐indole‐4‐carbonitrile (362 mg, 2.13 mmol) in DMF (19 mL) stirring at 0 °C was added sodium hydride (96 mg, 2.40 mmol, 60 % dispersion in mineral oil). The reaction was stirred for 30 min at 0 °C, then 1‐(chloromethyl)‐4‐methylbenzene (0.40 mL, 3.00 mmol) was added and the reaction stirred a further 18 h at ambient temperature. The mixture was diluted with saturated aqueous NH_4_Cl (40 mL) and partitioned between EtOAc (50 mL) and water (60 mL). The aqueous layer was extracted with EtOAc (2×) and the combined organic layers dried (MgSO_4_) and concentrated *in vacuo*. Purification by flash chromatography using 0–60 % EtOAc in heptane gave the title compound as an orange solid of 79 % purity (440 mg, 1.27 mmol, 60 %). ^1^H NMR (400 MHz, Chloroform‐d): δ 10.59 (s, 1H), 8.02 (s, 1H), 7.66 (d, *J=*7.5 Hz, 0.9 Hz, 1H), 7.61 (d, *J=*8.4 Hz, 0.9 Hz, 1H), 7.33 (t, *J=*8.0 Hz, 1H), 7.17 (d, *J=*7.7 Hz, 2H), 7.07 (d, *J=*8.0 Hz, 2H), 5.35 (s, 2H), 2.34 (s, 3H).

**1‐[(4‐Methylphenyl)methyl]‐3‐[(1E)‐2‐nitroethenyl]‐1H‐indole‐4‐carbonitrile (14)**

**13** (440 mg, 1.27 mmol, 79 % purity) and NH_4_OAc (350 mg, 4.54 mmol) were suspended in nitromethane (10 mL) and heated at 95 °C for 16 h. After cooling, the mixture was concentrated *in vacuo* and the residue partitioned between DCM (25 mL) and water (25 mL). The organic phase was isolated by hydrophobic frit and concentrated *in vacuo* to give the title compound as a brown solid of 93 % purity (284 mg, 0.83 mmol, 65 %). ^1^H NMR (400 MHz, Chloroform‐d): 8.81 (d, *J=*13.7 Hz, 1H), 7.70 (s, 1H), 7.65–7.57 (m, 3H), 7.33 (dd, *J=*8.3, 7.5 Hz, 1H), 7.19 (d, *J=*7.8 Hz, 2H), 7.07 (d, *J=*8.2 Hz, 2H), 5.35 (s, 2H), 2.36 (s, 3H).

**1‐[(4‐Methylphenyl)methyl]‐3‐(2‐nitroethyl)‐1H‐indole‐4‐carbonitrile (15)**

To a solution of **14** (280 mg, 0.82 mmol, 93 % purity) in THF (6 mL) and MeOH (0.8 mL) stirring at 0 °C was added sodium borohydride (100 mg, 2.63 mmol). The reaction was stirred for 20 min at 0 °C then 2 h at ambient temperature. The mixture was diluted with water (5 mL), treated with 1 M aqueous HCl (1 mL) and filtered through Celite®. The filter cake was washed with DCM (5 mL) and this organic filtrate concentrated *in vacuo*. Purification by flash chromatography using 0–50 % EtOAc in heptane gave the title compound as an orange oil of 93 % purity (120 mg, 0.35 mmol, 43 %). ^1^H NMR (400 MHz, Chloroform‐d) δ 7.52–7.44 (m, 2H), 7.20 (t, *J=*7.9 Hz, 1H), 7.15 (s, 1H), 7.12 (d, *J=*7.9 Hz, 2H), 6.96 (d, *J=*7.0 Hz, 2H), 5.25 (s, 2H), 4.78 (t, *J=*6.3 Hz, 2H), 3.69 (t, *J=*6.3 Hz, 2H), 2.32 (s, 3H). LCMS (Method C) RT 1.09 min, *m/z=*320 [M+H]^+^.

**3‐(2‐Aminoethyl)‐1‐[(4‐methylphenyl)methyl]‐1H‐indole‐4‐carbonitrile (Row847)**

To a solution of **15** (52 mg, 0.15 mmol, 93 % purity) and ammonium hydrochloride (36 mg, 0.67 mmol) in propan‐2‐ol (2.8 mL) and water (0.6 mL) was added iron powder (63 mg, 1.13 mmol). The reaction was then heated to 85 °C and stirred for 30 min. After cooling, the reaction mixture was filtered through Celite® and the filter cake washed with propan‐2‐ol (20 mL). The filtrate was diluted with aqueous K_2_CO_3_ (3 mL) and extracted with EtOAc (3×). The combined organic layers were washed with brine (10 mL), dried (MgSO_4_) and concentrated *in vacuo*. Purification by flash chromatography (NH‐functionalized media) using 40–100 % EtOAc in heptane gave the title compound as a white solid of 90 % purity (6.2 mg, 0.019 mmol, 13 %). ^1^H NMR (400 MHz, Chloroform‐d) 7.47 (dd, *J=*8.4, 0.8 Hz, 1H), 7.45 (dd, *J=*7.4, 0.8 Hz, 1H), 7.17 (dd, *J=*8.4, 7.4 Hz, 1H), 7.14–7.09 (m, 3H), 6.98 (d, *J=*8.1 Hz, 2H), 5.26 (s, 2H), 3.13 (t, *J=*6.4 Hz, 2H), 3.06 (t, *J=*6.4 Hz, 2H), 2.32 (s, 3H), 1.26 (s, 2H). LCMS (Method E) RT 3.83 min, *m/z=*290 [M+H]^+^.

#### Synthesis of compounds Row86 and Row745(2)

**4‐[4‐(1‐Hydroxybutyl)phenyl]‐1,2‐dihydrophthalazin‐1‐one (Row86)**

To a solution of *n‐*butyllithium (0.87 mL, 1.39 mmol, 1.6 M in hexanes) in THF (5 mL) stirring at −78 °C was added a solution of *n‐*butyraldehyde (90 μL, 1.0 mmol). After 30 min, 4‐(4‐bromophenyl)‐2H‐phthalazin‐1‐one (**Row86 (I)**) (200 mg, 0.66 mmol) was added and the solution allowed to warm to ambient temperature over 18 h. The mixture was diluted with saturated aqueous NH_4_Cl (5 mL) and partitioned between EtOAc (50 mL) and water (50 mL). The aqueous layer was extracted with EtOAc (3×) and the combined organic layers dried (MgSO_4_) and concentrated *in vacuo*. Purification by flash chromatography using 10–100 % EtOAc in heptane, followed by preparative HPLC gave the title compound as a white solid (15 mg, 0.05 mmol, 8 %). ^1^H NMR (400 MHz, DMSO‐d6): δ 12.81 (s, 1H), 8.36–8.32 (m, 1H), 7.93–7.86 (m, 2H), 7.70–7.67 (m, 1H), 7.53 (d, *J=*8.3 Hz, 2H), 7.49 (d, *J=*8.1 Hz, 2H), 5.23 (d, *J=*4.3 Hz, 1H), 4.62 (m, 1H), 1.70–1.56 (m, 2H), 1.45–1.26 (m, 2H), 0.90 (t, *J=*7.5 Hz, 3H). LCMS (Method A) RT 0.79 min, *m/z=*295 [M+H]^+^.

**4‐{4‐[Hydroxy(2‐methylcyclopropyl)methyl]phenyl}‐1,2‐dihydrophthalazin‐1‐one (16)**

The title compound was prepared as a mixture of diastereomers in an analogous manner to compound **Row86** using 4‐(4‐bromophenyl)‐2H‐phthalazin‐1‐one (**Row86 (I)**) and 2‐methylcyclopropane‐1‐carbaldehyde. ^1^H NMR (400 MHz, Chloroform‐d): δ 10.06 (s, 1H), 8.55–8.49 (m, 1H), 7.85–7.71 (m, 3H), 7.67–7.53 (m, 4H), 4.17 (m, 1H), 1.98 (m, 1H), 1.33 (d, *J=*6.5 Hz, 1H), 1.19 (d, *J=*6.4 Hz, 1H), 1.16–1.03 (m, 3H), 0.75–0.52 (m, 1H), 0.51–0.27 (m, 1H). LCMS (Method A) RT 0.78 min, *m/z=*307 [M+H]^+^.]

**4‐[4‐(2‐Methylcyclopropanecarbonyl)phenyl]‐1,2‐dihydrophthalazin‐1‐one (Row745(2))**

Dess‐Martin periodinane (35 mg, 0.083 mmol) was added to a solution of **16** (20 mg, 0.065 mmol) in DCM (0.7 mL) and stirred 16 h at ambient temperature. The solution was diluted with saturated aqueous NaHCO_3_ (2 mL) then partitioned between DCM (3 mL) and water (2 mL). The organic phase was isolated by hydrophobic frit and concentrated *in vacuo*. Purification by flash chromatography using 10–100 % EtOAc in heptane gave the title compound as a white solid of 87 % purity (2.5 mg, 0.008 mmol, 13 %). ^1^H NMR (400 MHz, Chloroform‐d): δ 10.46 (s, 1H), 8.59–8.50 (m, 1H), 8.18–8.10 (m, 2H), 7.88–7.64 (m, 5H), 2.50–2.40 (m, 1H), 1.59–1.52 (m, 1H), 1.26 (d, *J=*5.9 Hz, 3H), 1.13 (d, *J=*6.0 Hz, 1H), 0.97 (m, 1H). LCMS (Method B) RT 3.11 min, *m/z=*305 [M+H]^+^.

### Biological methods

#### PARP1 enzymatic assay

PARP1 was expressed and purified as described in the literature [65]. Briefly, full‐length human PARP1 was produced in *E. coli* Rosetta2 (DE3). The cells were harvested by centrifugation, and re‐suspended in lysis buffer containing 50 mM HEPES pH 8.0, 500 mM NaCl, 10 % glycerol, 0.5 mM TCEP and 10 mM imidazole. The sample was lysed using sonication in the presence of DNAse1 and 3‐aminobenzamide, centrifuged at 18,000 rpm for 1 h and the supernatant was filtered. The clarified sample was loaded to a HiTrap^TM^ IMAC column (Cytiva), washed with lysis buffer, then with a similar buffer containing 1 M NaCl, and finally with lysis buffer containing 25 mM imidazole before eluting the protein with 500 mM imidazole. The eluted sample was then diluted to reduce NaCl concentration to 250 mM, and loaded to a HiTrap Heparin column (Cytiva). The column was washed with 50 mM HEPES, 250 mM NaCl, 10 % glycerol, 1 mM EDTA, 0.1 mM TCEP, pH 7.5. and the sample was eluted using a gradient with a similar buffer containing 1 M NaCl. Fractions were analyzed by SDS‐PAGE and those containing protein were pooled and concentrated before aliquoting and stored at −70 °C.

IC_50_ was determined using homogeneous NAD^+^ consumption assay [65, 66]. 5 nM PARP1 was incubated with each compound in log half dilution series for 30 min in 50 mM Tris pH 8.0, 5 mM MgCl2, 0.2 mg mL^−1^ BSA, 10 μg mL^−1^ activated DNA and 500 nM NAD^+^. The reactions were performed in quadruplicates and resulting IC_50_ curves were fitted to a sigmoidal dose‐response curve (four variables) using GraphPad Prism version 8.02. Each experiment was repeated three times from which pIC_50_±SEM was calculated. Compounds with IC_50_ above 1 μM were measured only once.

#### PAMPA methods

Each donor solution was prepared by diluting a solution of the corresponding compound (DMSO, 1 mM) with phosphate buffer (pH 7.4, 0.025 M) up to a final concentration of 500 μM. Filters were coated with 10 μL of 1 % dodecane solution of phosphatidylcholine or 5 μL of brain polar lipid solution (20 mg mL^−1^ 16 % CHCl_3_, 84 % dodecane) prepared from CHCl_3_ solution 10 % w/v, for intestinal permeability and BBB permeability, respectively. The donor solution (150 μL) was then added to each well of the filter plate. 300 μL of the solution (50 % DMSO in phosphate buffer) were added to each well of the acceptor plate. The sandwich plate was assembled and incubated for 5 h at room temperature. After the incubation time, plates were separated, samples were taken from both the donor and acceptor wells, and the compound concentration was measured by UV/LC–MS. All compounds were tested in three independent experiments. Apparent permeability (P_app_) and membrane retention were calculated as described in Equation (3) and 4.

(3) $$P_{app}=\;\frac{V_{D}\times V_{A}}{\left(V_{D}+\;V_{A}\right)At}-ln\left(1-r\right)$$

Equation (3): Experimental apparent permeability (P_app_) calculation as described in the literature modified to yield values in cm s^−1^ [67, 68]. V_A_ is the volume in the acceptor well, V_D_ is the volume in the donor well (cm^3^), A is the effective area of the membrane (cm^2^), t is the incubation time (s) and r the ratio between drug concentration in the acceptor and equilibrium concentration of the drug in the total volume (V_D_+V_A_). Drug concentration is estimated by using the peak area integration.

(4) $$\%MR=\;\frac{\left[r-\left(D+A\right)\right]}{Eq}\;\times 100$$

Equation (4): Membrane retention calculation: r is the ratio between drug concentration in the acceptor and equilibrium solutions. D, A, and Eq represent the drug concentration in the donor, acceptor, and equilibrium solutions, respectively.

## AUTHOR CONTRIBUTIONS

G. M. G. designed the experiment, implemented the approach, and wrote the manuscript, S. F. synthesised the compounds, M. G. N. and G. M. assessed the quality of the samples from the synthesis, A. G. P. carried out the PARP1 enzymatic assay, L. L. managed and coordinated the execution of the PARP1 enzymatic assay, O. T. contributed to designing the experiments and writing the manuscript, A. B. carried out the PAMPA assay, J. W., D. H., J. E. A. W. contributed to the development of the reaction vector approach, M. J. B., B. C., V. J. G. supervised the project, V. J. G. also contributed to the writing of the manuscript. All authors contributed to the refinement of the manuscript.

## SUPPORTING INFORMATION

Additional supporting information can be found online in the Supporting Information section at the end of this article.

## Conflict of interests

G. M. G. participated in this project while he was a doctoral student at the Information School of the University of Sheffield during which time all of the compounds in this study were designed. Since completing his studies, G. M. G. has become a shareholder in AstraZeneca. The other authors declare no competing interests. For the purpose of open access, the author has applied a Creative Commons Attribution (CC BY) licence to any Author Accepted Manuscript version arising.

1

## Supporting information

As a service to our authors and readers, this journal provides supporting information supplied by the authors. Such materials are peer reviewed and may be re‐organized for online delivery, but are not copy‐edited or typeset. Technical support issues arising from supporting information (other than missing files) should be addressed to the authors.

Supporting Information

## Data Availability

Data sharing is not applicable to this article as no new data were created or analyzed in this study.
