# Supplementary material for: Synthetically accessible de novo design using reaction vectors: Application to PARP1 inhibitors
Source: Mol Inform. 2024 Feb 6;43(4):e202300183. doi: 10.1002/minf.202300183 (PMC11475289; doi:10.1002/minf.202300183)
Supplement: Supplementary file 1 — Supporting Information [file MINF-43-e202300183-s001.pdf]

molecular informatics

Supporting Information

Synthetically accessible de novo design using reaction vectors: Application to PARP1 inhibitors

Gian Marco Ghiandoni[1] | Stuart R. Flanagan[2] | Michael J. Bodkin[2] | Maria Giulia Nizi[4] | Albert Galera-Prat[5] | Annalaura Brai[6] | Beining Chen[3] | James E. A. Wallace[2] | Dimitar Hristozov[2] | James Webster[1] | Giuseppe Manfroni[4] | Lari Lehtiö[5] | Oriana Tabarrini[4] | Valerie J. Gillet[1]

Supplementary Information

1. Machine Learning models - descriptors and average performance metrics (15 validations per model)

PARP1 activity regression

Molecular Descriptor	R^2	MAE	MSE
Avalon - 1024 bits	0.73 (± 0.02)	0.46 (± 0.03)	0.42 (± 0.04)
Binary FeatMorgan (Radius 2) - 1024 bits	0.74 (± 0.02)	0.46 (± 0.02)	0.41 (± 0.04)
Count FeatMorgan (Radius 2) - 1024 bits	0.75 (± 0.02)	0.46 (± 0.03)	0.40 (± 0.04)
Binary Morgan (Radius 2) - 1024 bits	0.74 (± 0.02)	0.46 (± 0.03)	0.40 (± 0.04)
Count Morgan (Radius 2) - 1024 bits	0.75 (± 0.01)	0.45 (± 0.03)	0.39 (± 0.04)
RDKit - 1024 bits	0.73 (± 0.02)	0.47 (± 0.02)	0.43 (± 0.04)
2D Descriptors - 185 bits	0.66 (± 0.03)	0.54 (± 0.03)	0.54 (± 0.06)

Pgp and BCRP substrate and BBB classifications

	Pgp Substrate				BCRP Substrate				BBB Penetration			
Descriptor	Recall	Precision	F1-score	MCC	Recall	Precision	F1-score	MCC	Recall	Precision	F1-score	MCC
Avalon (Binary)	0.70	0.70	0.70	0.40	0.73	0.74	0.72	0.43	0.93	0.94	0.93	0.80
FeatMorgan (Binary)	0.72	0.72	0.72	0.44	0.66	0.65	0.63	0.25	0.92	0.92	0.92	0.77
FeatMorgan (Count)	0.74	0.75	0.74	0.48	0.64	0.64	0.61	0.22	0.93	0.93	0.93	0.78
Morgan (Binary)	0.67	0.68	0.66	0.34	0.68	0.68	0.66	0.31	0.92	0.93	0.92	0.77
Morgan (Count)	0.69	0.71	0.69	0.40	0.69	0.71	0.67	0.35	0.93	0.93	0.92	0.77
MOE Descriptors	0.77	0.78	0.77	0.55	0.82	0.82	0.82	0.62	0.92	0.92	0.92	0.78

Molecular descriptors

Method	Type	Description
Avalon	Fingerprint	Substructure fingerprint that hashes paths and features of molecules using a set of definitions. It was originally developed as part of the Avalon Chemoinformatics Toolkit. The implementation is similar to that of the Daylight fingerprint.
FeatMorgan	Fingerprint	Similarity fingerprint that hashes circular atom environments up to a specified bond radius. The fingerprint represents molecules as pharmacophores by encoding their chemical features (e.g., acidic, basic, hydrogen-bond donor/acceptor) and connectivity.
Morgan	Fingerprint	Similarity fingerprint that hashes circular atom environments up to a specified bond radius. The fingerprint encodes atom types, number of heavy neighbours, number of hydrogens, charges, isotopes, and connectivity. Morgan is also referred to as extended-connectivity fingerprint (ECFP).
RDKit	Fingerprint	Substructure fingerprint that hashes linear and branched molecular subgraphs by combining atom and bond features. The implementation is similar to that of the Daylight fingerprint.
Atom/Bond Counts	Descriptor	Counts of the total number of atoms/bonds in a molecule.
BCUT	Descriptor	Eigenvalue-based descriptors calculated from the weighted version of the Burden matrix of a molecule. The Burden matrix describes both the connectivity and atomic properties of the molecule. The eigenvalues of the matrix are then extracted to yield a small set of orthogonal descriptors. (BCUT stands for Burden, Chemical-abstracts-service, and University of Texas)
Chi and Kappa	Descriptor	Chi Connectivity Indices encode weighted counts of substructure fragments from hydrogen-suppressed molecular graphs. The indices can capture structural features including size, cycles, branching, bond orders, and heteroatoms. Kappa Shape Indices aim to encode the shape of molecules from hydrogen-suppressed molecular graphs into three values (Kappa values). The values are derived from the counts of one-bond, two-bond and three-bond fragments in a molecule.
GCUT	Descriptor	Eigenvalue-based descriptors calculated from a modified adjacency matrix of a molecule. The matrix describes both the connectivity and atomic properties of the molecule. The eigenvalues of the matrix are then extracted to yield a small set of orthogonal descriptors.
SlogP	Descriptor	Calculated logP of a molecule from the sum of its atomic contributions to lipophilicity. The method uses SMARTS definitions to retrieve the atomic contributions.
SMR	Descriptor	Calculated molecular refractivity of a molecule from the sum of its atomic contributions. The method uses SMARTS definitions to retrieve the atomic contributions.
VSA	Descriptor	Approximate surface area of a molecule from the sum of the sphere areas of its atoms. Spheres are calculated using van der Waals radii and atomic distances.

2. GOLD docking model

Docking parameters

- autoscale=2
- radius=10
- save_lone_pairs=0
- early_termination=0
- docking_fitfunc_path=plp
- rescore_fitfunc_path=goldscore

Docking results - validation scores and numbers of consistent poses per ligand

Query	Mean PLP.Fitness	Mean GoldScore.Fitness	Pose Consistency
Olaparib	91.87	51.16	8/10
Rucaparib	88.57	62.82	10/10
(3S)-Niraparib	96.02	50.45	10/10
(3R)-Niraparib	85.97	57.12	N.A.
(11S,12R)-Talazoparib	83.54	47.01	10/10
(11R,12S)-Talazoparib	66.08	46.56	N.A.
(11S,12S)-Talazoparib	68.70	55.61	N.A.
(11R,12R)-Talazoparib	80.69	59.49	N.A.
PJ34	86.98	53.67	10/10

Notes: The ligands extracted from the crystallographic data are highlighted in grey, while their virtually generated stereoisomers are in white. The pose consistency of the stereoisomers is marked with not applicable as no crystallographic data is available for those structures.

3. BRICS fragmentation

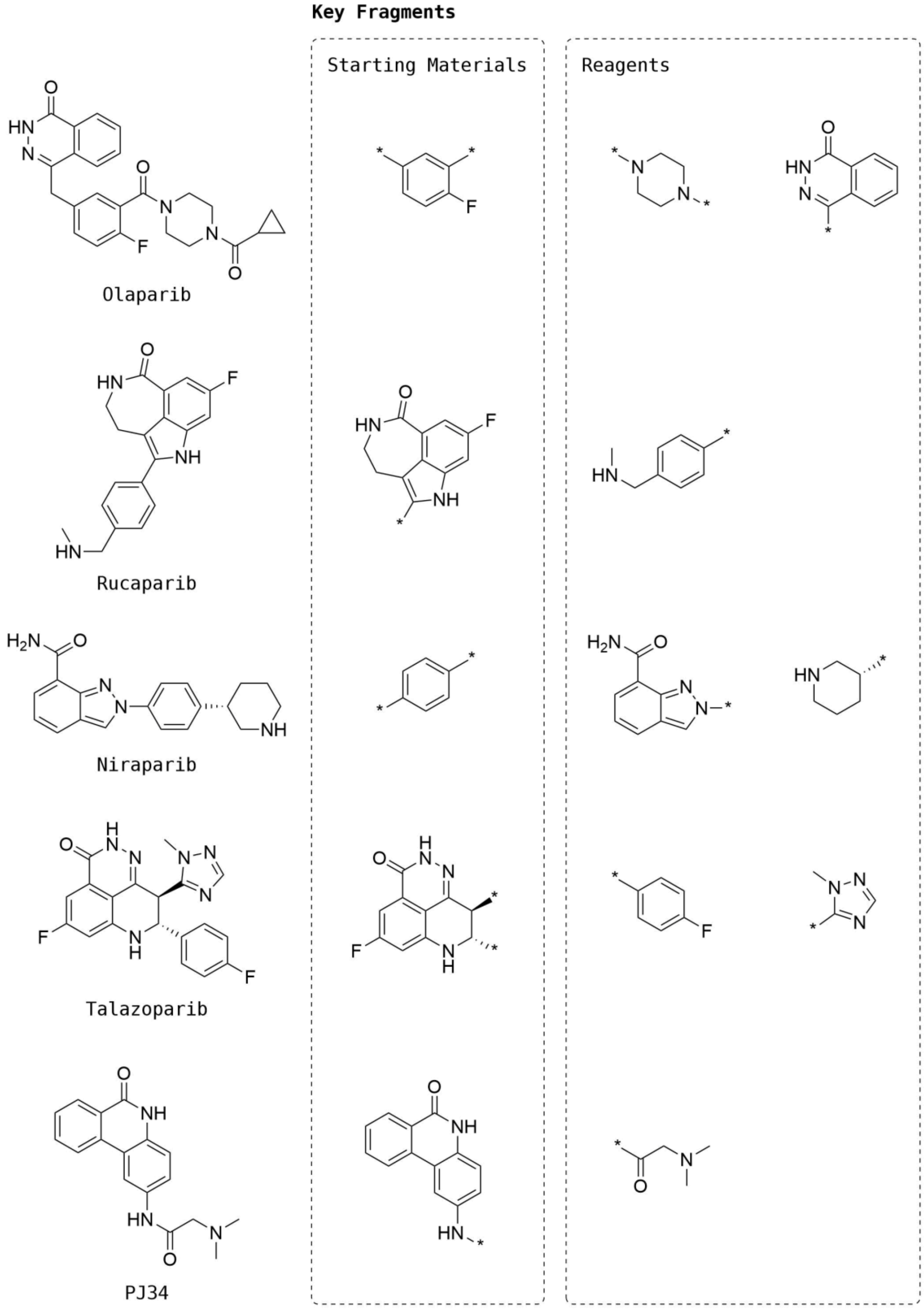

4. RENATE parameters

Query	Experiment #	Parameters
Olaparib	1	minFragmentSize=1, MaxStartingMaterials=30, MaxReagents=25000, NumProductsCycle=2000, numFinalProducts=1000
Rucaparib	1	minFragmentSize=5, MaxStartingMaterials=30, MaxReagents=25000, NumProductsCycle=2000, numFinalProducts=1000
Rucaparib	2	minFragmentSize=5, MaxStartingMaterials=750, MaxReagents=1000, NumProductsCycle=4000, numFinalProducts=1000
Niraparib	1	minFragmentSize=5, MaxStartingMaterials=30, MaxReagents=25000, NumProductsCycle=2000, numFinalProducts=1000
Niraparib	2	minFragmentSize=5, MaxStartingMaterials=750, MaxReagents=1000, NumProductsCycle=4000, numFinalProducts=1000
Talazoparib	1	minFragmentSize=1, MaxStartingMaterials=750, MaxReagents=1000, NumProductsCycle=4000, numFinalProducts=1000
PJ34	1	minFragmentSize=5, MaxStartingMaterials=750, MaxReagents=1000, NumProductsCycle=4000, numFinalProducts=1000

5. Candidate structures for synthesis and their scores

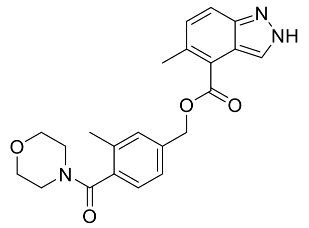	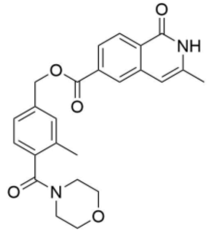	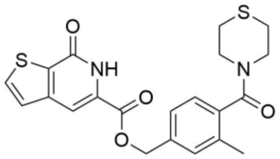	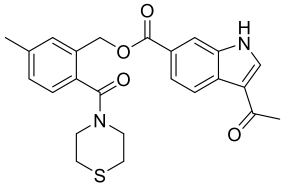	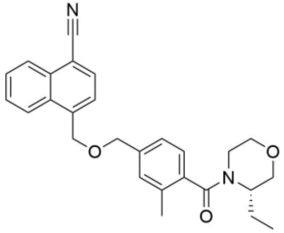
Olaparib - Row26	Olaparib - Row514	Olaparib - Row217	Olaparib - Row563	Olaparib - Row538
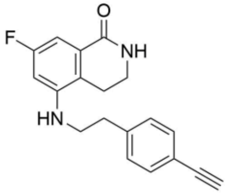	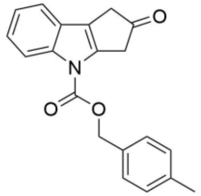	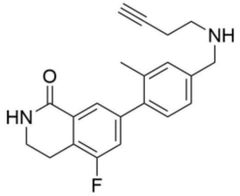	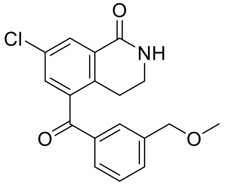	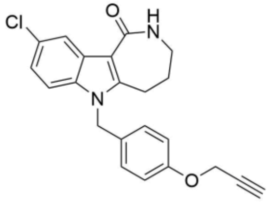
Rucaparib - Row312	Rucaparib - Row443	Rucaparib - Row528	Rucaparib - Row665	Rucaparib - Row600
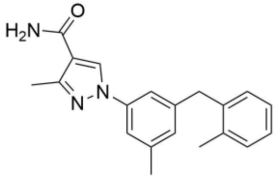	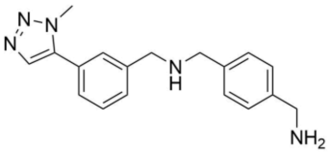	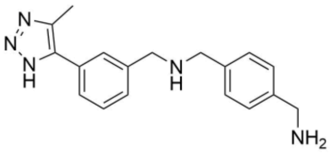	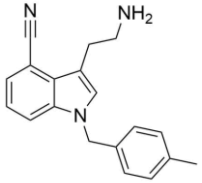	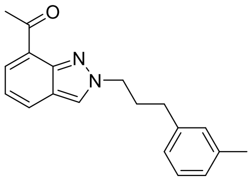
Niraparib - Row113	Niraparib - Row760	Niraparib - Row760c	Niraparib - Row847	Niraparib - Row408

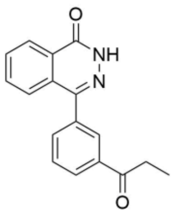	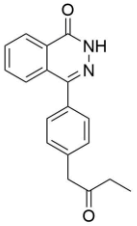	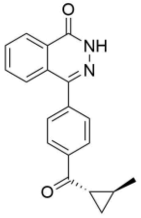	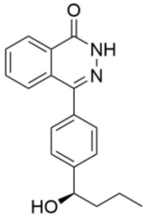	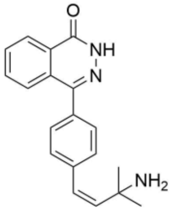
PJ34 - Row27	PJ34 - Row5	PJ34 - Row745(2)	PJ34 - Row86	PJ34 - Row304

Query	Candidate	PLP.Fitness (Mean ± SD)	Goldscore.Fitness (Mean ± SD)	Pose Consistency	PARP1 QSAR pIC50 (uM)
Olaparib	Row26	88.96 ± 1.96	61.06 ± 2.09	10/10	0.92
Olaparib	Row514	90.33 ± 3.28	53.76 ± 2.78	6/10	0.79
Olaparib	Row217	84.81 ± 6.55	59.65 ± 6.17	4/10	0.84
Olaparib	Row563	90.09 ± 2.68	56.73 ± 1.89	4/10	0.78
Olaparib	Row538	93.04 ± 3.92	45.40 ± 12.33	6/10	0.78
Rucaparib	Row312	85.60 ± 1.52	51.30 ± 0.20	3/10	0.70
Rucaparib	Row443	81.01 ± 1.08	53.80 ± 2.77	8/10	0.64
Rucaparib	Row528	87.42 ± 1.83	49.79 ± 2.72	10/10	0.66
Rucaparib	Row665	74.62 ± 0.40	33.78 ± 6.74	3/10	0.59
Rucaparib	Row600	84.62 ± 4.04	43.96 ± 0.93	2/10	0.66
Niraparib	Row113	86.70 ± 0.34	58.50 ± 2.43	8/10	0.86
Niraparib	Row760	87.27 ± 1.50	40.96 ± 16.88	7/10	0.71
Niraparib	Row760c	78.50 ± 1.65	49.94 ± 5.91	6/10	0.49
Niraparib	Row847	88.95 ± 0.93	60.48 ± 4.45	6/10	0.58
Niraparib	Row408	87.39 ± 2.45	55.45 ± 6.59	7/10	0.78
PJ34	Row27	88.65 ± 0.21	55.33 ± 1.64	10/10	0.74
PJ34	Row5	85.35 ± 1.56	57.54 ± 1.81	9/10	0.84
PJ34	Row745(2)	80.76 ± 1.11	61.37 ± 1.04	7/10	0.47
PJ34	Row86	86.80 ± 0.29	53.61 ± 2.77	8/10	0.66
PJ34	Row304	95.14 ± 2.18	70.46 ± 5.42	6/10	0.56

6. Synthetic routes - proposed by RENATE and adjusted by the chemists

Notes: Additional steps introduced by the chemists are highlighted in dashed squares. Precursors and intermediates selected for the biological assay are identified with the notation “(I)”.

Olaparib - Row26

Proposed Route:

Step 1
Ref: US6900227B2

Step 2
Ref: US4112096

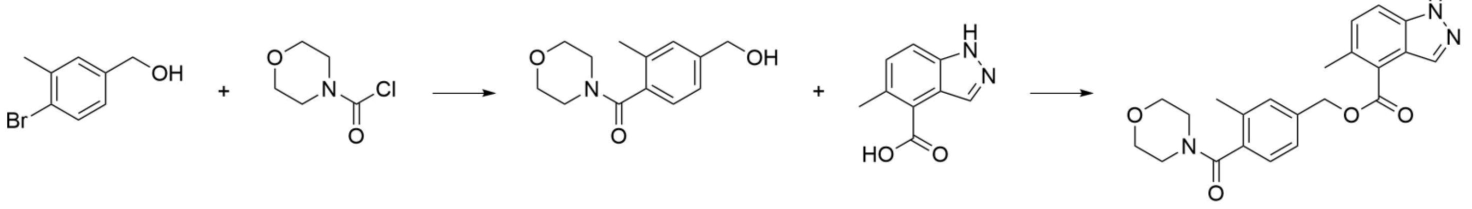

Row26

Actual Route:

Protection **Step 1** **Deprotection**

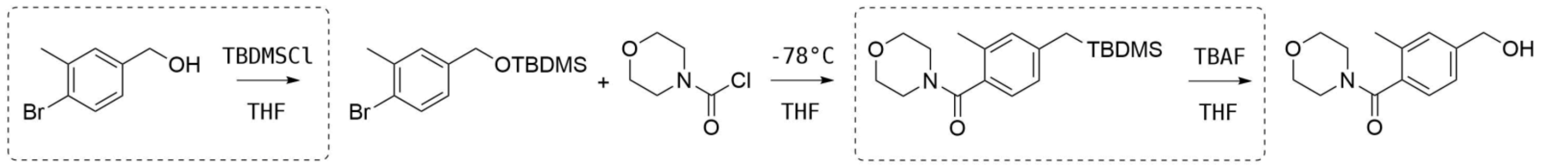

Step 2

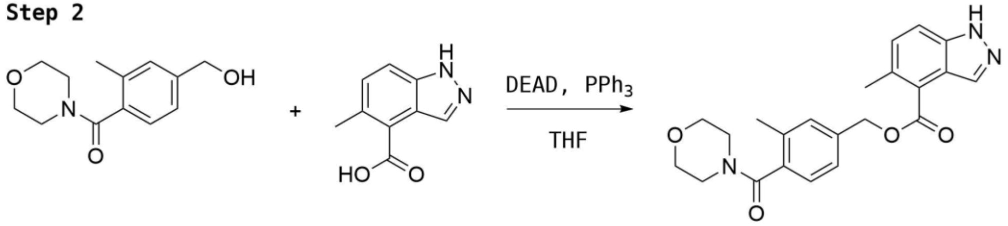

Olaparib - Row514

Proposed Route:

Step 1
Ref: US6900227B2

Step 2
Ref: US8476253B2

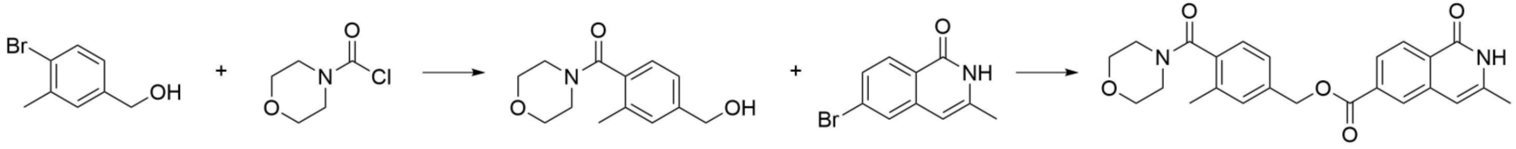

Row514

Actual Route:

Protection

Step 1

Deprotection

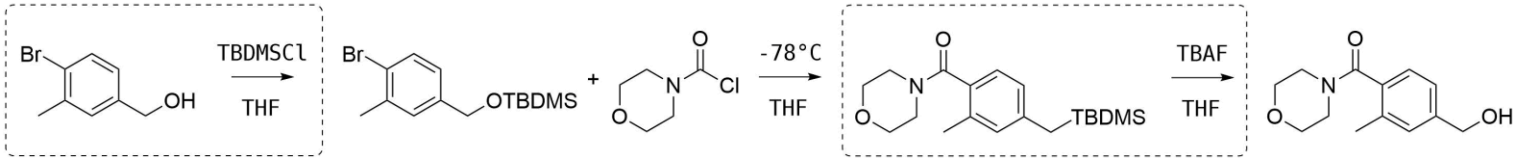

Group Conversion

Step 2

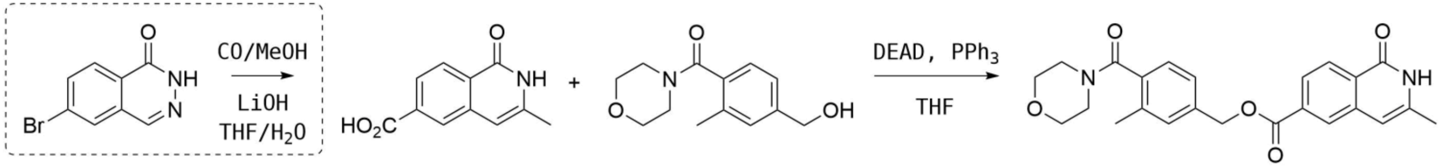

Rucaparib - Row528

Proposed Route:

Step 1
Ref: US5380910

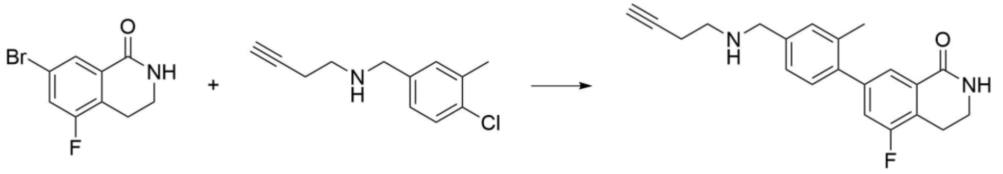

Row528

Actual Route:

Building Block Conversion

Suzuki Coupling (Alternative to Ullmann-type Coupling)

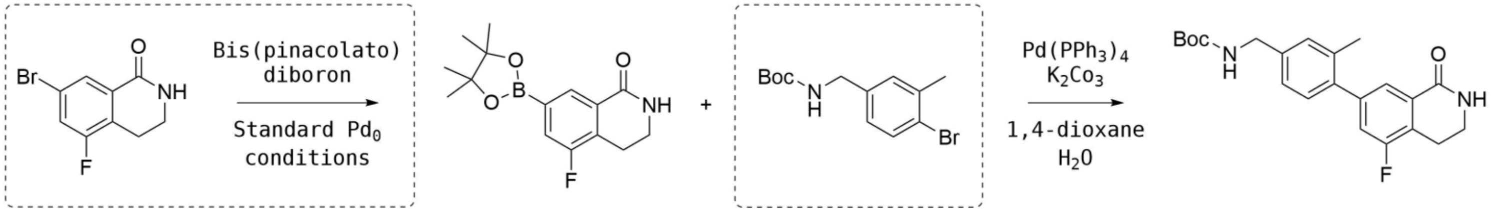

Functionalisation

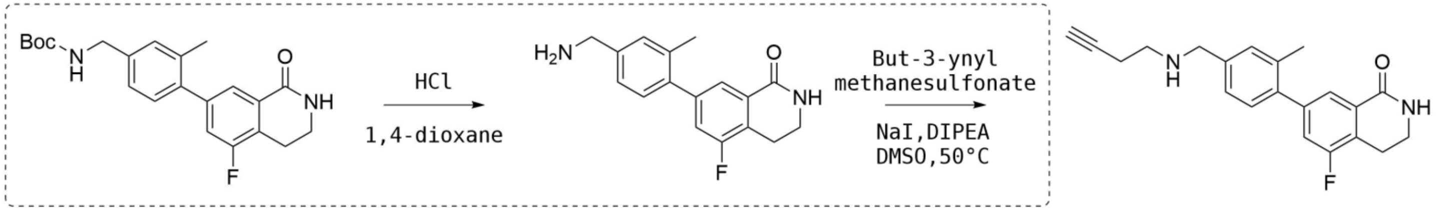

Row528 (I)

Niraparib - Row847

Proposed Route:

Step 1
Ref: US4104467

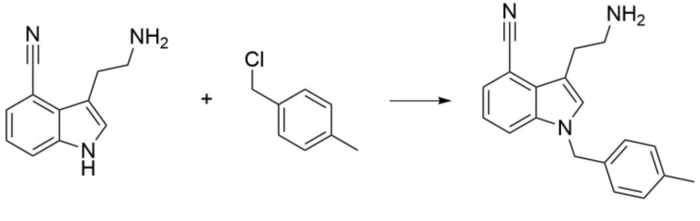

Row847

Actual Route:

Step 1

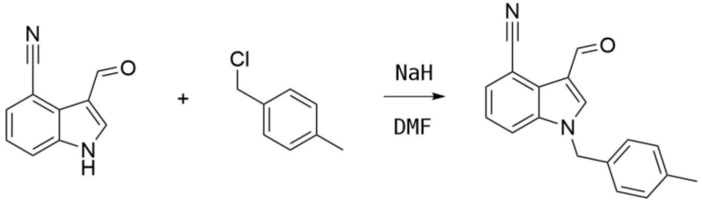

Functionalisation

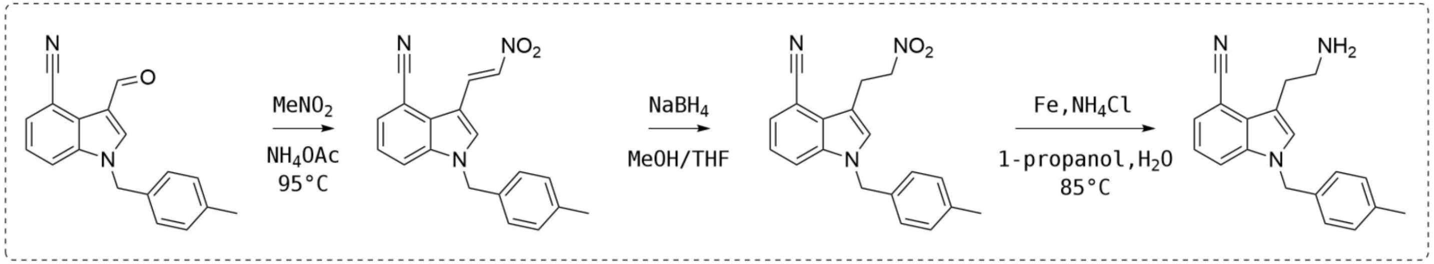

PJ34 - Row745(2)

Proposed Route:

Step 1
Ref: US4028404

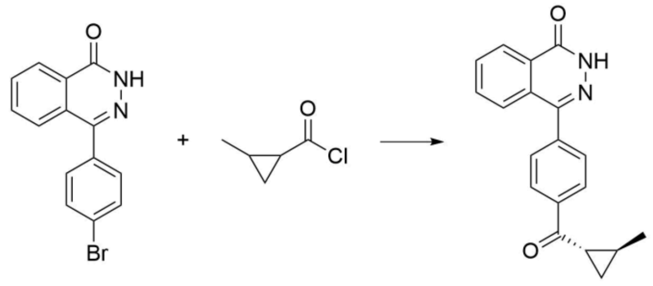

Row745(2)

Actual Route:

Organolithium Alternative

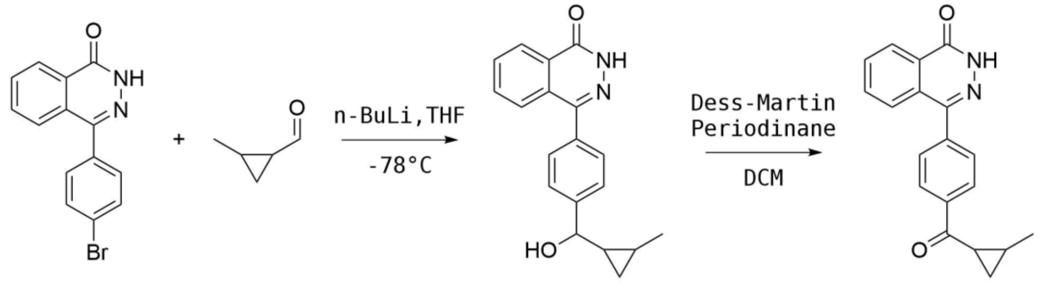

Row86 (I)
(Row745(2) (I))

PJ34 - Row86

Proposed Route:

Step 1
Ref: US5156763

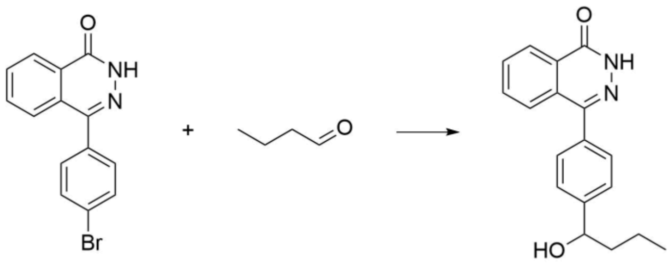

Row86

Actual Route:

Organolithium Alternative

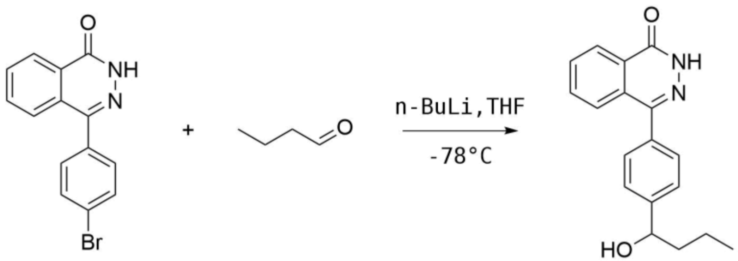

Row86 (I)
